# Supplementary material for: Fragility of surface states in topological superfluid 3He
Source: Nat Commun. 2021 Mar 10;12:1574. doi: 10.1038/s41467-021-21831-y (PMC7946958; doi:10.1038/s41467-021-21831-y)
Supplement: Supplementary file 1 — Supplementary Information [file 41467_2021_21831_MOESM1_ESM.pdf]

Supplementary Information: Fragility of surface states in topological superfluid ^{3}He

P. J. Heikkinen,[1,*] A. Casey,[1] L. V. Levitin,[1] X. Rojas,[1] A. Vorontsov,[2]
P. Sharma,[3] N. Zhelev,[4] J. M. Parpia,[4] and J. Saunders[1]

[1]*Department of Physics, Royal Holloway, University of London, Egham, Surrey, TW20 0EX, UK*
[2]*Department of Physics, Montana State University, Bozeman, Montana 59717, USA*
[3]*Department of Physics, Indian Institute of Science, Bangalore 560 012, India*
[4]*Department of Physics, Cornell University, Ithaca, NY 14853, USA*

CONTENTS

Supplementary Fig. 1: Setup for NMR measurements

Supplementary Fig. 2: FEM simulation of distortion of the cavity due to overpressure

Supplementary Note 1: Connection between frequency shifts and energy gap

- **Supplementary Fig. 3:** Determination of initial slopes in the cavity
- **Supplementary Fig. 4:** Comparison of measured initial slopes to earlier results and theory

Supplementary Note 2: Bulk marker frequency shifts and temperature gradient across cavity

- **Supplementary Fig. 5:** Bulk marker frequency shifts
- **Supplementary Fig. 6:** Temperature gradient and temperature hysteresis across the cavity

Supplementary Note 3: Theoretical calculation of the order parameter in ^{3}He slab geometry

- **Supplementary Fig. 7:** Spatial dependence of energy gap in slab-shaped cavity

Supplementary Note 4: Strong-coupling corrections

- **Supplementary Fig. 8:** Strong-coupling corrections to bulk energy gap
- **Supplementary Fig. 9:** Suppression of energy gap with "diffuse" boundary condition

Supplementary Note 5: Determination of T_c in presence of solid ^{3}He surface boundary layer

- **Supplementary Fig. 10:** Magnetisation with solid layer of ^{3}He on the cavity walls
- **Supplementary Fig. 11:** Superfluid frequency shift in the cavity with solid ^{3}He on the walls

Supplementary Note 6: Pair breaking at the surface

- **Supplementary Fig. 12:** Suppression of T_c by magnetic scattering on quantum spins

* petri.heikkinen@rhul.ac.uk

Supplementary Note 7: Temperature correction

Supplementary Fig. 13: Calibration of heating caused by NMR pulses

Supplementary Fig. 14: Model parameters used in temperature correction

Supplementary References [1]–[49]

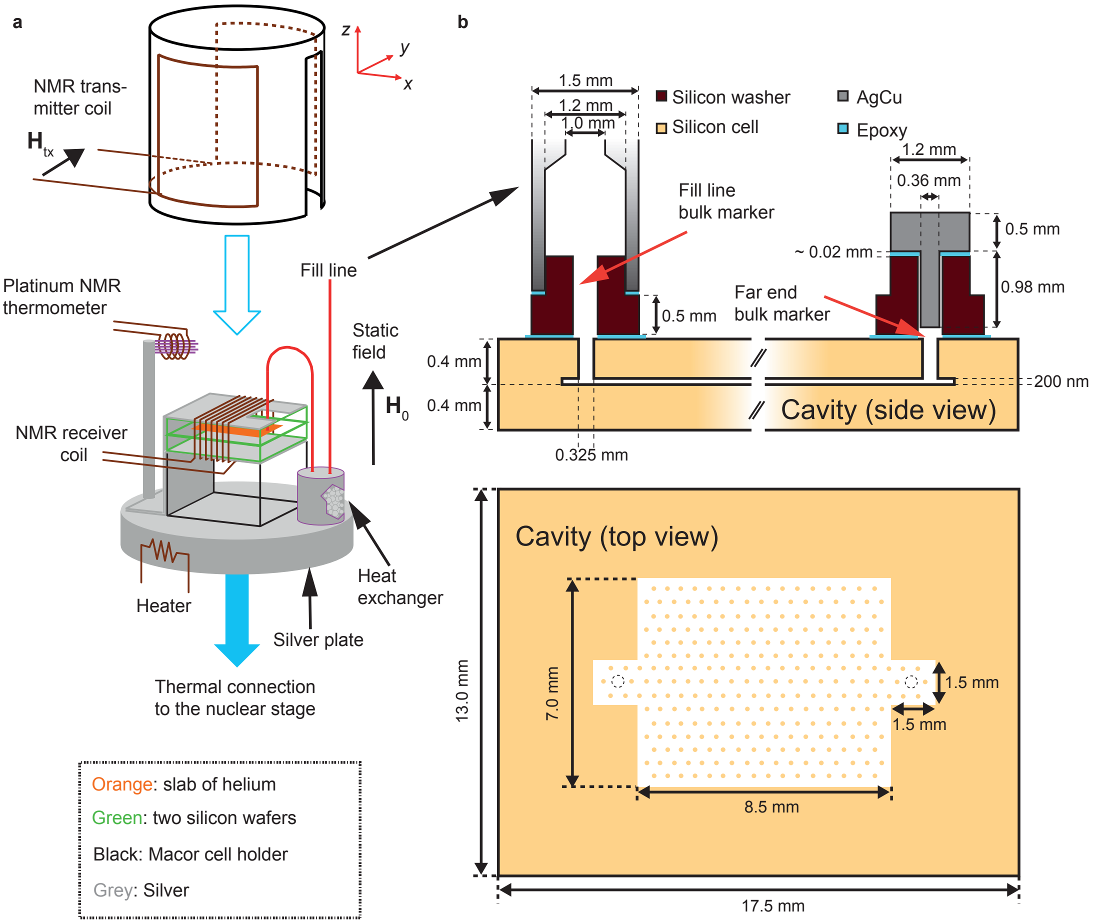

Supplementary Figure 1: Setup for NMR measurements. a Schematics of the NMR setup. Silicon-silicon sample container sits on a silver plate thermally connected to the copper nuclear stage of the cryostat. Silver foils glued on both sides of the silicon structure and screwed on the silver plate help to thermalise the sample container. Nanofabricated slab-shaped cavity is filled with helium through a sintered silver heat exchanger having surface area 8 m^2. ^{3}He in the cavity is cooled via the ^{3}He column in the fill line. Temperature of the silver plate is measured using a platinum NMR thermometer. Main NMR field $\mathbf{H}_0$ is created with a solenoid coil located inside the 4 K bath far from the sample region. Saddle-shaped transmitter coil used to create the NMR pulse field $\mathbf{H}_{\rm tx}$ is wound on sides of a Macor holder sliding around the sample region on the silver plate. The precessing sample magnetisation is measured with a receiver coil wound tightly around the sample container. **b** Dimensions of the sample container. Most of the volume in the fill line is shielded from NMR measurements by the metallic fill line. The small unshielded part on the bottom end as well as the small compartment on the far end of the cavity have volumes of the same order as the cavity to result in three comparable peaks in NMR spectra. Triangular lattice of 100 µm diameter pillars separated by 500 µm reduces the distortion of cavity due to liquid overpressure.

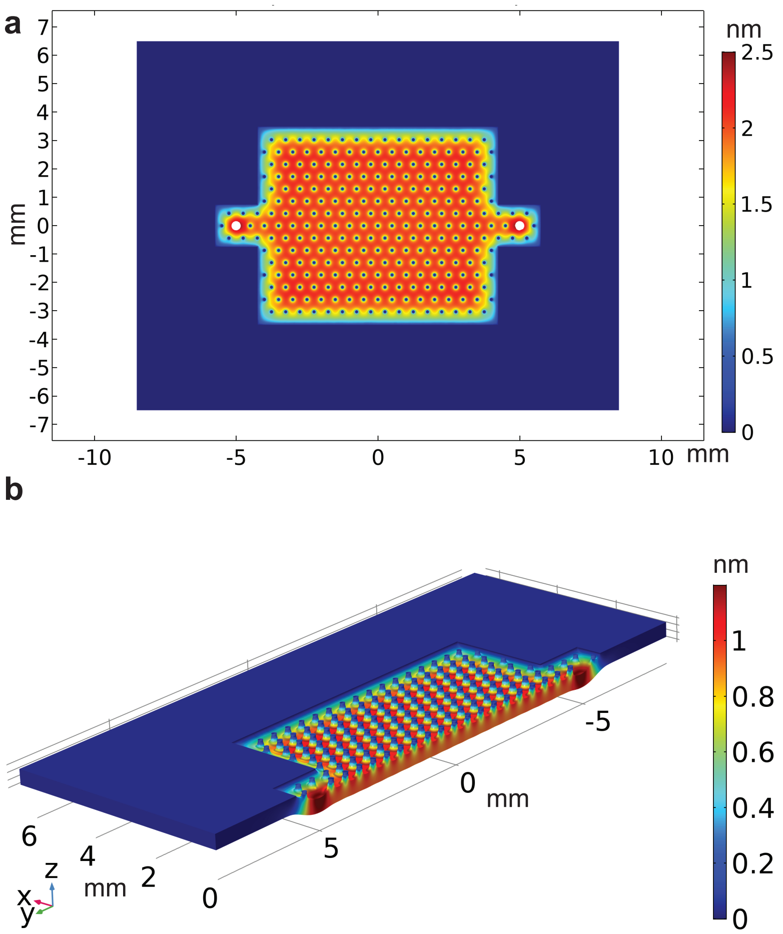

Supplementary Figure 2: FEM simulation of distortion of the cavity due to overpressure. a Change in the cavity height due to 1 bar internal pressure, as simulated with finite element method (FEM), using COMSOL Multiphysics, increases sharply when moving from the cavity edges towards the centre. Maximal simulated distortion with triangular lattice of support pillars in the centre region is $2.1\,\mathrm{nm{\cdot}bar^{-1}}$. We estimate a $0.5\,\mathrm{nm{\cdot}bar^{-1}}$ uncertainty in the simulation and thus use higher value $2.6\,\mathrm{nm{\cdot}bar^{-1}}$ for the maximal cavity height distortion in analysis. **b** Three-dimensional illustration of the internal cavity distortion. The effect has been greatly magnified here for clarity. We use the same material parameters for silicon as in Ref. [1]: density $\rho_{\mathrm{Si}} = 2329\,\mathrm{kg{\cdot}m^{-3}}$, the Young's modulus $E_{\mathrm{Si}} = 170\,\mathrm{GPa}$, and the Poisson's ratio $\nu_{\mathrm{Si}} = 0.28$.

SUPPLEMENTARY NOTE 1: Connection between frequency shifts and energy gap

Here we restrict the discussion to linear spin dynamics relevant to our NMR experiments with small spin tipping angles β. The following discussion covers the observed frequency shift in the cavity; the frequency shifts observed in the bulk marker volumes are discussed in Supplementary Note 2.

In normal ^{3}He the NMR precession occurs at the Larmor frequency $f_{\mathrm{L}} = \gamma H_0/2\pi$, where γ is the gyromagnetic ratio of ^{3}He. Frequency shifts $\Delta f_{\mathrm{sf}} = f - f_{\mathrm{L}}$ of different superfluid phases below T_{c} are determined by the curvature of dipole energy as a function of rotations in spin space [2]. Both positive and negative frequency shifts are possible depending on whether the dipole energy is at minimum or at maximum before the spin-tipping NMR pulse, respectively. In ^{3}He-A the order parameter, and thus the magnitude of the dipole energy, is parametrised with two unit vectors $\hat{\mathbf{l}}$ and $\hat{\mathbf{d}}$ exhibiting long-range order. Here $\hat{\mathbf{l}} \parallel \mathbf{L}$ describes the orientation of the orbital angular momentum of all the Cooper pairs, and the order-parameter vector $\mathbf{d}$ points in the direction of zero spin projection: $\hat{\mathbf{d}} \perp \mathbf{S}$. In general, the directions of both vectors are determined by a competition between orienting effects including external magnetic field, boundaries of sample, dipole energy, and superfluid flow. Applied magnetic field H_0 larger than dipolar field $H_{\mathrm{D}} \sim 5\,\mathrm{mT}$ locks $\hat{\mathbf{d}} \perp \mathbf{H}_0$, whereas the dipole energy is minimised when $\hat{\mathbf{l}} \parallel \hat{\mathbf{d}}$. Any surface introduces strong boundary condition $\hat{\mathbf{l}} \parallel \hat{\mathbf{s}}$ where $\hat{\mathbf{s}}$ is normal to the surface. The length scale over which boundaries affect the order parameter is determined by the dipolar length $\xi_{\mathrm{D}} \sim 10\,\mu\mathrm{m}$ [2].

We have $\mathbf{H}_0 \parallel \hat{\mathbf{z}}$ and $\hat{\mathbf{s}} \parallel \hat{\mathbf{z}}$. The magnetic field defines the orientation $\hat{\mathbf{d}} \perp \hat{\mathbf{z}}$ and the small height of the cavity ($D \ll \xi_{\mathrm{D}}$) forces $\hat{\mathbf{l}} \parallel \hat{\mathbf{z}}$ everywhere in it. This dipole-unlocked configuration maximises the dipole energy, resulting in a negative NMR frequency shift in the A phase [3, 4]:

$$f^2 - f_{\mathrm{L}}^2 = -\frac{\gamma^2 \lambda_{\mathrm{D}} N_{\mathrm{F}}}{5\pi^2 \chi_{\mathrm{N}}} \Delta_{\mathrm{A}}^2 = -\left(\frac{\Omega_{\mathrm{A}}}{2\pi}\right)^2 . \tag{1}$$

Here Ω_{A} is the A-phase Leggett frequency (the longitudinal resonance frequency) depending on both the temperature and the pressure, and energy gap Δ_{A} refers to the maximum A-phase energy gap in the momentum space at $\hat{\mathbf{p}} \perp \hat{\mathbf{l}}$. Here $\lambda_{\mathrm{D}} \sim 10^{-6}$ sets the relative scale of the dipole energy, N_{F} is the density of states at the Fermi level, and χ_{N} is the normal state spin susceptibility equalling the susceptibility of equal-spin-paired ^{3}He-A. All pre-factors of Δ_{A}^2 depend only on the normal state properties of ^{3}He independent of the level of confinement or boundary condition. Thus, all the dependences of the cavity frequency shift at constant pressure are fully defined by the energy gap.

In a cavity with non-specular quasiparticle scattering at the walls, the gap acquires a spatially inhomogeneous suppression $\Delta_{\mathrm{A}}(z)$ as seen in Supplementary Fig. 7. However, the NMR precession in a confinement volume where $D \ll \xi_{\mathrm{D}}$ is uniform with the frequency shift [4, 5]

$$\left|f^2 - f_{\mathrm{L}}^2\right| = \frac{\gamma^2 \lambda_{\mathrm{D}} N_{\mathrm{F}}}{5\pi^2 \chi_{\mathrm{N}}} \left\langle \Delta_{\mathrm{A}}^2(z) \right\rangle , \tag{2}$$

where $\left\langle \Delta_{\mathrm{A}}^2(z) \right\rangle$ refers to the spatially averaged value of the squared energy gap, the experimental determination of which we now discuss.

For compactness we write $\left|f^2 - f_{\mathrm{L}}^2\right| = \zeta \left\langle \Delta_{\mathrm{A}}^2(z) \right\rangle$, where $\zeta = \frac{\gamma^2 \lambda_{\mathrm{D}} N_{\mathrm{F}}}{5\pi^2 \chi_{\mathrm{N}}}$ is a pressure-dependent, temperature-independent constant. In bulk in the Ginzburg-Landau (G-L) regime, near bulk transition temperature T_{c0}, $\Delta_{\mathrm{A}}^2 \propto (1 - T/T_{\mathrm{c0}})$. Therefore, in this regime, we define $\mathrm{IS}_{\Delta}^{\mathrm{bulk}}$ and

$\mathrm{IS}^{\mathrm{bulk}}$ by

$$\left|f^2 - f_{\mathrm{L}}^2\right| = \zeta \mathrm{IS}_{\Delta}^{\mathrm{bulk}} \left(1 - \frac{T}{T_{\mathrm{c0}}}\right) = \mathrm{IS}^{\mathrm{bulk}} \left(1 - \frac{T}{T_{\mathrm{c0}}}\right). \tag{3}$$

Now clearly $\zeta = \mathrm{IS}^{\mathrm{bulk}}/\mathrm{IS}_{\Delta}^{\mathrm{bulk}}$.

For our "specular" ($S = 0.98$) surface, which shows essentially no T_{c} suppression, the measured frequency shift corresponds to the spatially uniform and unsuppressed bulk gap Δ_{A}, Supplementary Eq. (1). Thus, we have for the precession frequency in the cavity

$$\left|f^2 - f_{\mathrm{L}}^2\right| = \mathrm{IS}_{\mathrm{slab}}^{'\mathrm{spec}'} \left(1 - \frac{T}{T_{\mathrm{c}}}\right), \tag{4}$$

where $T_{\mathrm{c}} = T_{\mathrm{c0}}$ to a good approximation. Here $\mathrm{IS}_{\mathrm{slab}}^{'\mathrm{spec}'} \approx \mathrm{IS}^{\mathrm{bulk}}$ is determined as a slope of a linear fit between the cavity frequency shift and temperature close enough to T_{c} where the dependence is expected to be nearly linear [4]:

$$\mathrm{IS}_{\mathrm{slab}}^{'\mathrm{spec}'} = \frac{\partial \left|f^2 - f_{\mathrm{L}}^2\right|}{\partial \left(1 - T/T_{\mathrm{c}}\right)}, \qquad \text{averaged over } 0.90 T_{\mathrm{c}} < T < T_{\mathrm{c}}. \tag{5}$$

Similarly, $\mathrm{IS}_{\Delta}^{\mathrm{bulk}}$ is determined from a linear fit to the calculated bulk gap over equivalent temperature range:

$$\mathrm{IS}_{\Delta}^{\mathrm{bulk}} = \frac{\partial \Delta_{\mathrm{A}}^2}{\partial \left(1 - T/T_{\mathrm{c0}}\right)}, \qquad \text{averaged over } 0.90 T_{\mathrm{c0}} < T < T_{\mathrm{c0}}. \tag{6}$$

The choice of this temperature range is a suitable compromise between precision and accuracy and justifies calling the proportionality constants IS as initial slopes, see Supplementary Fig. 3.

Determination of constant ζ using the ratio of the two abovementioned initial slopes provides a high degree of cancellation of the systematic error arising from the choice of temperature range. This is true for both weak-coupling and strong-coupling models in the G-L regime (Supplementary Note 4). Now we get

$$\left\langle \Delta_{\mathrm{A}}^2(z) \right\rangle = \frac{\mathrm{IS}_{\Delta}^{\mathrm{bulk}}}{\mathrm{IS}_{\mathrm{slab}}^{'\mathrm{spec}'}} \left|f^2 - f_{\mathrm{L}}^2\right|. \tag{7}$$

This expression determines the average gap suppression for arbitrary surface scattering at all temperatures. The procedure described here eliminates any systematic errors that might arise from the use of literature values of the bulk frequency shift. To compare the experimental and theory-based specular and non-specular initial slopes we can use the dependence:

$$\frac{\mathrm{IS}_{\mathrm{slab}}^{'\mathrm{diff}'}}{\mathrm{IS}_{\mathrm{slab}}^{'\mathrm{spec}'}} = \frac{\mathrm{IS}_{\Delta}^{'\mathrm{diff}'}}{\mathrm{IS}_{\Delta}^{\mathrm{bulk}}}, \tag{8}$$

where the superscript 'diff' refers to any non-specular boundary condition. This dependence holds as long as the suppressed gap $\left\langle \Delta_{\mathrm{A}}^2(z) \right\rangle = \mathrm{IS}_{\Delta}^{'\mathrm{diff}'} \left(1 - T/T_{\mathrm{c}}\right)$.

Supplementary Fig. 4a shows the comparison between the measured values of the initial slopes and the earlier experimental values from Refs. [6–8] in the pressure range covered in the current experiments. Since the previous experiment with largest pressure overlap with us used approximately 5% range below T_{c0} ($0.95 T_{\mathrm{c0}} < T < T_{\mathrm{c0}}$) to determine the bulk initial slopes [6, 7], we extract the corresponding values from our "specular" measurements for direct comparison

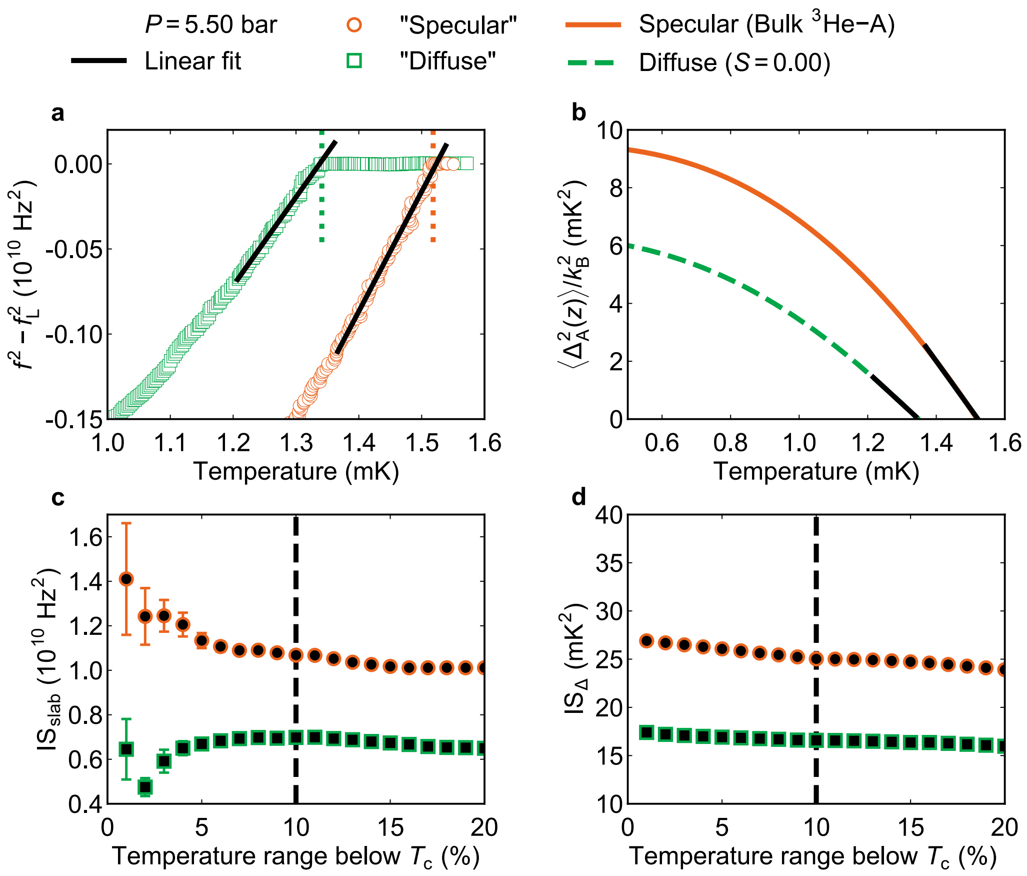

Supplementary Figure 3: Determination of initial slopes in the cavity. a Measured frequency shift $f^2 - f_L^2$ against temperature T with "specular" (circles) and "diffuse" (squares) boundary conditions at pressure $P = 5.50$ bar, showing the linear fits over the temperature range $0.90T_c < T < T_c$ (solid black lines) used to determine the experimental initial slopes $\mathrm{IS_{slab}}$. Vertical dotted lines indicate the measured superfluid transition temperature in the cavity, T_c. **b** Calculated spatially averaged values of the squared quasiclassical weak-coupling energy gap with specular (solid orange curve) and diffuse (dashed green curve) boundary conditions. Linear fits over the range $0.90T_c < T < T_c$ (solid black lines) are used to determine the theory-based initial slopes $\mathrm{IS_\Delta}$. **c** Initial slopes $\mathrm{IS_{slab}}$ as a function of temperature range of the linear fit below measured T_c for "specular" (circles) and "diffuse" (squares) boundary conditions. Increased uncertainty limits and scatter approaching T_c arise from limited amount of data combined with noise, temperature gradient across the cavity (Supplementary Fig. 6), and potential rounding of T_c due to cavity height distortion (Supplementary Fig. 2). **d** Initial slopes $\mathrm{IS_\Delta}$ as a function of temperature range of the linear fit below calculated T_c for specular (circles) and diffuse (squares) boundary conditions. We estimate the systematic error between the actual initial slope and the slope determined over the range $0.90T_c < T < T_c$ to be 8%. The range chosen for conversion between the frequency shift and the energy gap, Supplementary Eq. (7), is marked by vertical dashed line in **c** and **d**.

instead of using the 10% range defined above. This eliminates the difference between systematic errors. As expected, the measured initial slopes, $\mathrm{IS}_{\mathrm{slab}}^{'\mathrm{spec}'}$, compare well with the values obtained in bulk ^{3}He-A, $\mathrm{IS}_{\mathrm{A}}^{\mathrm{bulk}}$. The linear fit given in Ref. [9] to original data from Refs. [6, 7] is $\mathrm{IS}_{\mathrm{A}}^{\mathrm{bulk}} = \left(0.479 + 0.109\,\mathrm{bar}^{-1}P\right)\cdot 10^{10}\,\mathrm{Hz}^2$, whereas the fit to current experiments using the equivalent 5% range is $\mathrm{IS}_{\mathrm{slab}}^{'\mathrm{spec}'} = \left(0.436 + 0.131\,\mathrm{bar}^{-1}P\right)\cdot 10^{10}\,\mathrm{Hz}^2$. The earlier fit is based on data taken up to 22 bar, so higher pressure experiments under specular confinement would be required to see whether any significant difference persists or whether the seen difference between the fits is due to scatter in the experimental values.

The initial slopes determined for "diffuse" boundary condition are compared to theory in Supplementary Fig. 4b. We see that our experiments agree well with specularity $S = 0.10$, further confirming that to be the best value of specularity for the boundaries with 32 µmol·m^{-2} ^{4}He preplating.

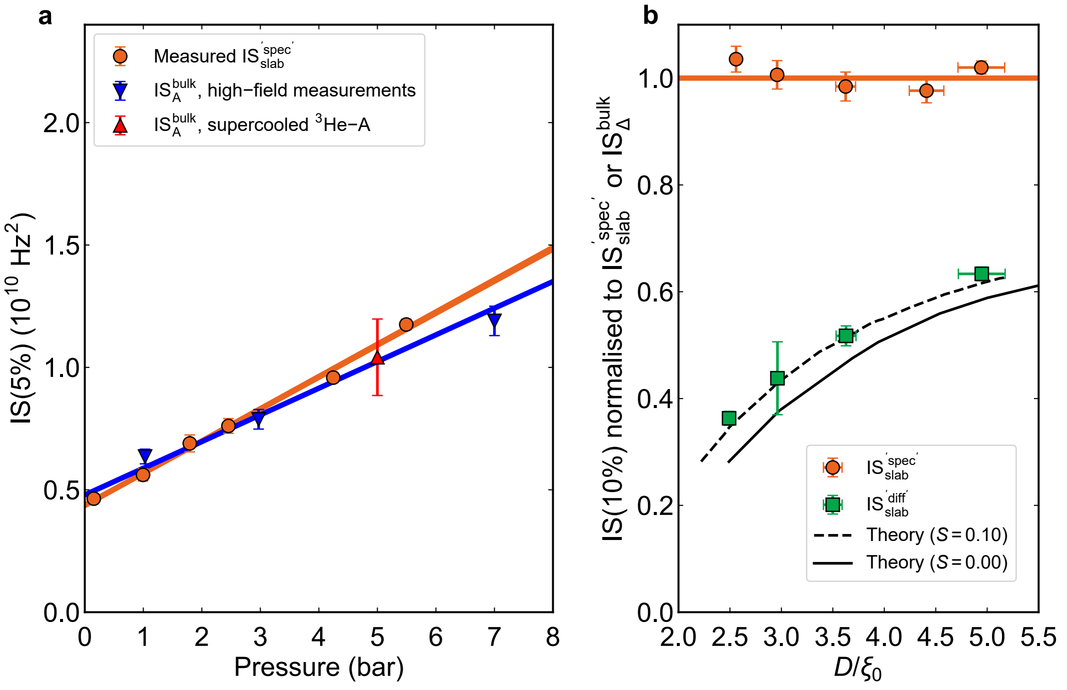

Supplementary Figure 4: Comparison of measured initial slopes to earlier results and theory. a Measured initial slopes $IS_{slab}^{'spec'}$ (orange circles, 5% range below T_c) in cavity with "specular" boundary condition show a linear low-pressure dependence (orange line) similar to the previous experimental data. Blue line is a linear fit given in Ref. [9] based on IS_A^{bulk} measured using transverse NMR frequency shifts in bulk ^{3}He-A at pressures below polycritical point (21.22 bar) at high magnetic fields (blue downward-pointing triangles) [6, 7]. In Ref. [8] (red upward-pointing triangle) the frequency shifts were measured by supercooling bulk A phase significantly below its stable field-pressure configuration in the phase diagram. They determined initial slopes over the temperature range $0.90T_{c0} < T < T_{c0}$, so here we scale their data into the 5% range, using the estimated difference in the systematic errors (see Supplementary Fig. 3d). **b** Initial slopes for "diffuse" scattering (squares) normalised to measured "specular" initial slopes (circles) are compared to quasiclassical weak-coupling theory giving $IS_\Delta^{'diff'}/IS_\Delta^{bulk}$ (black lines, see Supplementary Eq. (8)) in the range $0.90T_c < T < T_c$. The data agree best with curve for specularity $S = 0.10$ (dashed line). Vertical error bars represent the experimental uncertainty in determination of the initial slopes and horizontal error bars in **b** show the modelled spread of cavity height distortion by pressure (Supplementary Fig. 2).

SUPPLEMENTARY NOTE 2: Bulk marker frequency shifts and temperature gradient across cavity

Helium in the bulk liquid compartments on both ends of the cavity can nucleate either into ^{3}He-B or ^{3}He-A at bulk superfluid transition temperature T_{c0}. Supercooling of the A phase is more likely in the far-end bulk compartment which is isolated from the rest of the system via nanofluidic cavity. The frequency shift in bulk ^{3}He-A, which is at minimum of dipole energy, has the same magnitude but opposite sign compared to frequency shift in the cavity, Supplementary Eq. (1). The bulk B-phase frequency shift is

$$f_{\mathrm{B}}^2 - f_{\mathrm{L}}^2 = \frac{3\gamma^2\lambda_{\mathrm{D}}N_{\mathrm{F}}}{4\pi^2\chi_{\mathrm{B}}}\Delta_{\mathrm{B}}^2\sin^2\beta_n = \left(\frac{\Omega_{\mathrm{B}}}{2\pi}\right)^2\sin^2\beta_n, \tag{9}$$

where Ω_{B} is the B-phase Leggett frequency, Δ_{B} is the isotropic B-phase energy gap, χ_{B} is the temperature-dependent B-phase spin susceptibility, and β_n is the angle between $\mathbf{H}_0$ and spin-orbit rotation axis $\hat{\mathbf{n}}$ [2]. The preferred orientation in bulk is such that $\beta_n = 0$. However, at vertical walls of the bulk marker compartments, dipolar energy forces $\beta_n \approx 63.4°$ leading to $\sin^2\beta_n = 0.8$ and to positive frequency shift from Larmor value detected at superfluid transitions. Since the frequency shifts in our experiments are small compared to the Larmor frequency, we use correspondence $f^2 - f_{\mathrm{L}}^2 \approx 2f_{\mathrm{L}}\Delta f$ for a straightforward conversion between calculated energy gap and frequency shift.

Examples of detected frequency shifts against temperature are presented in Supplementary Fig. 5 and in Fig. 1b,c in the main text. The wall-determined value of B-phase frequency shift is the dominant one in the fill-line bulk marker while the supercooled A phase dominates the far-end bulk marker. Tracking of bulk marker frequency shifts to the lowest temperatures has not been successful, since their amplitudes rapidly decrease below T_{c0} and vanish into noise at $T \approx 0.9T_{c0}$. In the B phase a temperature-dependent drop in amplitude is expected due to both decreasing value of χ_{B} and increasing value of Ω_{B}, which together with a smooth bending of $\hat{\mathbf{n}}$ between wall-favoured and field-favoured orientations in a macroscopic compartment results in spectral broadening of the signal [2]. However, the detected drop is much faster than expected and neither of these effects concerns the A phase. In the usual experiments, where the cavity signal is of the highest interest, a field gradient along the z axis, used to separate the bulk marker signals from the cavity signal, causes additional broadening, but this effect is independent of temperature. Even with optimized bulk marker signal detection — after removing the field gradient along the z axis and diminishing the cavity signal by applying a field gradient on the xy plane — the bulk marker amplitudes drop below detection level soon below $T = 0.9T_{c0}$. The reason for this remains unknown.

Possible temperature gradient across the cavity is seen as different measured temperatures of the silver plate, T_{Ag}, during the superfluid transitions in the bulk volumes. These two silver-plate temperatures are denoted by $T_{\mathrm{c}}^{\mathrm{fill}}$ and $T_{\mathrm{c}}^{\mathrm{far}}$. After the correction for the thermal gradient across the heat exchanger (Supplementary Note 7), $T_{\mathrm{c}}^{\mathrm{fill}} \approx T_{\mathrm{c}}^{\mathrm{G}}$. The small measured temperature gradient as a function of pressure and temperature hysteresis as a function of temperature sweep rate are presented in Supplementary Fig. 6.

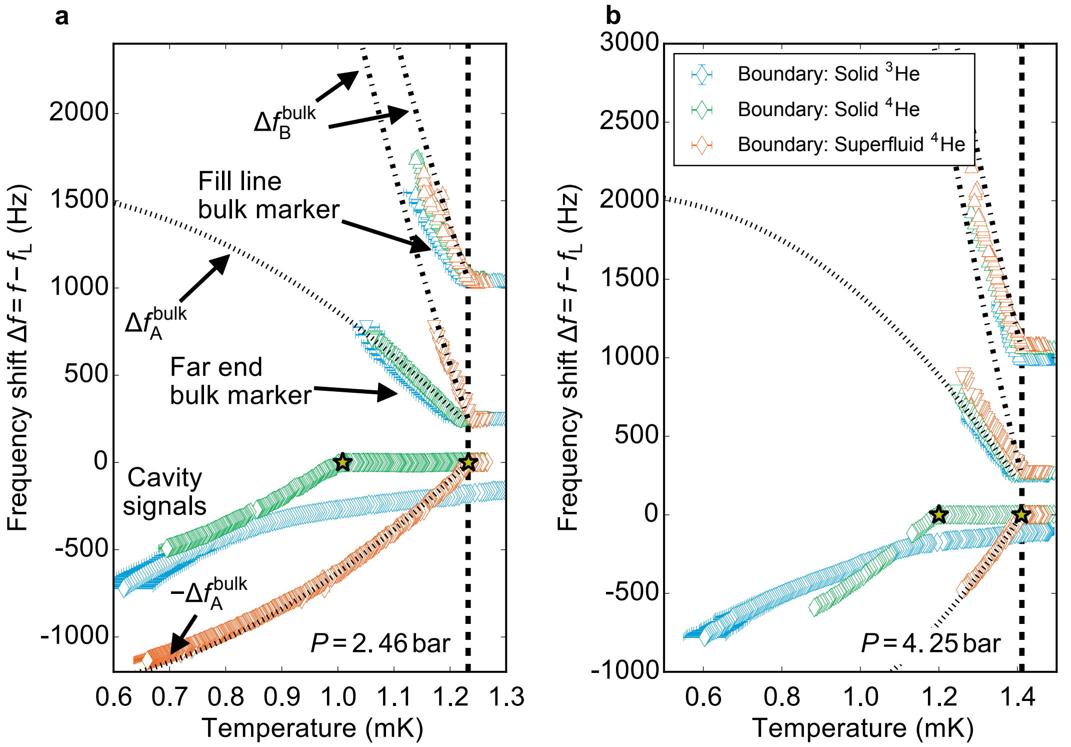

Supplementary Figure 5: Bulk marker frequency shifts. **a** Composite of measured frequency shifts (symbols) of both fill line and far end bulk markers with cavity frequency shifts at 2.46 bar. The fill line bulk marker always shows the B phase. The far end bulk marker can remain in the supercooled A phase to the lowest temperatures at which signal is detected. Vertical dashed line indicates the literature value of bulk superfluid transition, T_c^G [10]. Calculated bulk A-phase frequency shifts Δf_A^{bulk} (dotted lines) are converted from weak-coupling energy gap Δ_A, using Supplementary Eq. (7) and measured “specular” initial slopes, whereas bulk B-phase frequency shifts Δf_B^{bulk} (dash-dotted lines, wall-determined value, Supplementary Eq. (9)) are directly based on energy gap Δ_B including temperature-dependent trivial strong-coupling corrections [11, 12], using parameter library in Ref. [13]. Stars mark the easily-identifiable “specular” and “diffuse” superfluid transitions in the cavity. **b** Equivalent data at 4.25 bar. Horizontal error bars indicate the uncertainty in temperature determination (see Supplementary Note 7).

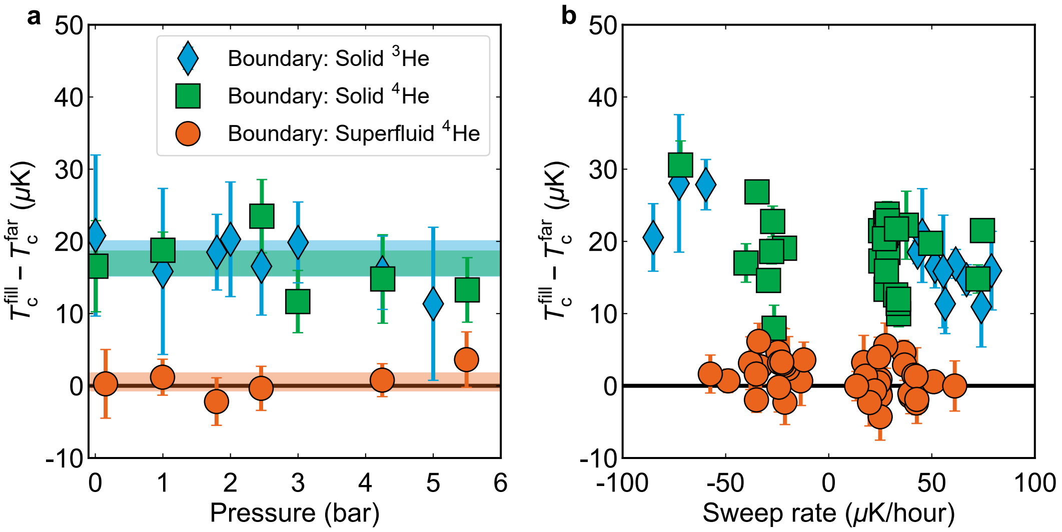

Supplementary Figure 6: Temperature gradient and temperature hysteresis across the cavity. **a** Temperature difference across the cavity at bulk superfluid transition temperature T_{c0}, $\Delta T_{\mathrm{cavity}} = T_c^{\mathrm{fill}} - T_c^{\mathrm{far}}$. With close to specular boundary condition (circles) the gradient across the cavity is practically zero at all pressures, whereas with solid ^{3}He (diamonds) or ^{4}He (squares) on the surface the gradient is measured to be around 20 µK. Each symbol represents an average of all temperature sweeps conducted at a single pressure with error bars indicating the experimental uncertainty. The coloured bands show the values averaged over all pressures for each boundary condition. **b** The temperature hysteresis in the cell turns out to be insignificant as characterised by the difference between the measured bulk marker transition temperatures as a function of temperature sweep rate. Most of the measurements have been conducted at absolute sweep rates less than 50 µK·h^{-1}.

SUPPLEMENTARY NOTE 3: Theoretical calculation of the order parameter in ^{3}He slab geometry

The theoretical procedure to obtain the spatial profile of the order parameter and the corresponding density of states (DOS) in the cavity is based on the quasiclassical theory of superfluid ^{3}He [11]. The relevant details for slab geometry can be found in many publications, see for example Refs. [14, 15].

The multi-component order parameter is determined self-consistently together with the quasiclassical quasiparticle correlator (Green's function) $\widehat{g}(\mathbf{R}, \mathbf{k}; \epsilon_m)$, which describes propagation of quasiparticles with energy ϵ_m along straight classical trajectories. In cavities thinner than $D \sim 10\xi_0$, not only the symmetry of the order parameter, but also the nature of the quasiparticle scattering on surfaces determine the behaviour of the quantum condensate. The spectrum of the sub-gap states in the vicinity of the surface is different depending on whether the scattering is specular, diffuse, retroreflecting [16] (maximally pair-breaking in odd-parity superfluid), or a mixture of these, resulting in the different suppression of both the order parameter and the transition temperature.

The boundary conditions for the quasiclassical propagator at the surface encode the scattering processes with high momentum transfer $\delta k \sim k_{\rm F}$. We adopt the random $\mathcal{S}$-matrix scattering model [17–19] with unitary scattering matrix for particle-like excitations,

$$\mathcal{S}_{qq'} = -\frac{1 - i\eta}{1 + i\eta}\Big|_{qq'}, \tag{10}$$

where $\{q, q'\}$ are in-plane vectors, and the scattering is from q'(in) to q(out). For hole-like excitations one has $\tilde{\mathcal{S}}_{q'q} = \mathcal{S}^*_{qq'}$ (final and initial states exchanged), and the entire particle-hole space is covered by

$$\widehat{\mathcal{S}} = \begin{pmatrix} \mathcal{S} & 0 \\ 0 & \tilde{\mathcal{S}}^\dagger \end{pmatrix}. \tag{11}$$

Random Hermitian matrix η has properties

$$\overline{\eta_{qq'}} = 0, \qquad \overline{\eta^*_{qq'}\eta_{kk'}} = \kappa(q - q')\delta_{q-q',k-k'}, \tag{12}$$

where overline refers to statistical average. The correlation cumulant is taken as a constant, $\kappa(q - q') = 2W/\sum_q 1$ [18]. Matrix η depends on a particular surface scattering picture and for example can be expressed in terms of parameters of the randomly rippled wall model [17].

The $\mathcal{S}$-matrix model allows a continuous description of surface scattering from specular to fully diffuse limit by tuning the W-parameter, which we further generalize to include retroreflection by multiplying $\mathcal{S}$ by the overall retro delta function $\delta_{-q,q'}$. The amplitude of the specular reflection is given as

$$\overline{\mathcal{S}_{kp}} = -\delta_{kp}\frac{1 - \sigma}{1 + \sigma} \tag{13}$$

with surface self-energy σ determined from self-consistency equation $\sigma = \overline{\eta\frac{1}{1+\sigma}\eta} = \frac{2W}{1+\sigma}$. This allows one to define 'specularity', i.e., the probability of specular scattering, of the surface as $S = \pm(1 - \sigma)^2/(1 + \sigma)^2$. The negative sign indicates retroreflection. For $W = 0$ one has $\sigma = 0$ with either completely specular $S = +1$ or retroreflective $S = -1$ surface; for $W = 1$ we get $\sigma = 1$ and fully diffuse surface $S = 0$ with suppressed $T_{\rm c}(D)$ that exactly follows [18] the original result for diffuse $T_{\rm c}$ suppression given by Kjäldman *et al.* (KKR) [20].

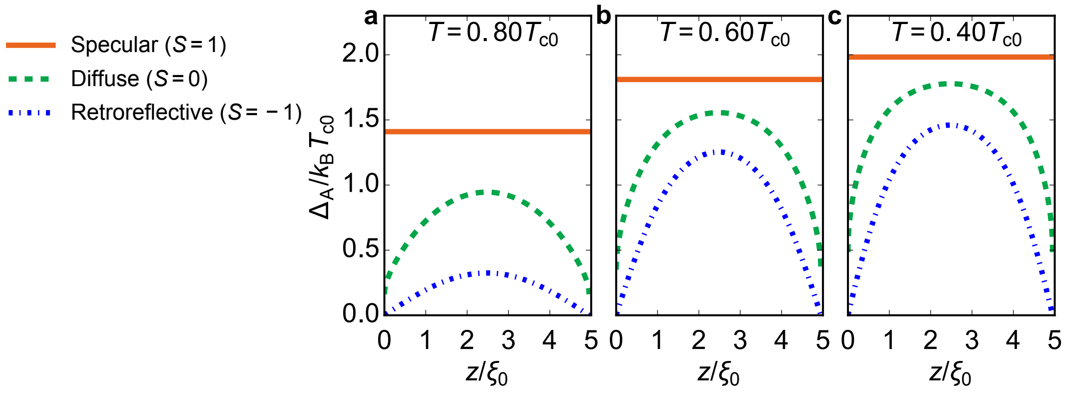

Supplementary Figure 7: Spatial dependence of energy gap in slab-shaped cavity. With specular boundary condition (solid orange lines) for quasiparticles, the energy gap Δ_A remains constant equalling the corresponding bulk value. With diffuse boundary condition (dashed green lines), the gap is suppressed at the walls resulting in spatial dependence along the z-axis, whereas maximally pair-breaking retroreflective boundary condition (dash-dotted blue lines) fully suppresses the gap at the walls. The values shown here at three different temperatures T (**a, b, c**), where T_{c0} is the bulk superfluid transition temperature, correspond to effective cavity height $D/\xi_0 = 4.95$ ($P = 5.50$ bar in our cavity) and are based on quasiclassical weak-coupling theory for superfluid ^{3}He [11], following the calculational methods presented in the text.

The boundary condition that connects the propagators of the incoming ($\mathbf{p}_{\rm in}$) and outgoing ($\mathbf{p}_{\rm out}$) trajectories at the surface is written as [19]

$$\widehat{A}\widehat{g}(\mathbf{p}_{\rm out}) = \widehat{g}(\mathbf{p}_{\rm in})\widehat{A}, \qquad \widehat{A} = \frac{1 + i\widehat{\sigma}}{1 - i\widehat{\sigma}}. \tag{14}$$

The surface self-energy $\widehat{\sigma}$ is momentum-independent and computed self-consistently from

$$\widehat{\sigma} = \frac{2W}{1 + \widehat{\sigma}^2}\left(\widehat{g}_{\rm surf} - \widehat{\sigma}\right). \tag{15}$$

Here the surface propagator for specular (or retro) reflection is averaged over the momenta parallel to surface, which we can write as integration over incoming or outgoing momenta on the Fermi surface (FS)

$$\widehat{g}_{\rm surf} = \langle \widehat{g}_{\rm in} \rangle_{||} = \langle \widehat{g}_{\rm out} \rangle_{||} = \langle |\hat{v}_z| \widehat{g}_{\rm in} \rangle_{\rm FS-} = \langle |\hat{v}_z| \widehat{g}_{\rm out} \rangle_{\rm FS+}. \tag{16}$$

The integral is normalised with the area of FS in the reflective plain, πp_F^2, i.e.,

$$\langle \dots \rangle_{||} = \frac{1}{\pi p_F^2} \iint dp_x dp_y \cdots = \frac{1}{\pi} \int_{\hat{v}_z > 0 \text{ or } \hat{v}_z < 0} d\Omega_F |\hat{v}_z| \dots \tag{17}$$

In the normal state $\widehat{g}_{\rm surf} = \widehat{g}_N = -i\,\mathrm{sgn}(\epsilon_n)\widehat{\tau}_3$, and solution for surface self-energy is $\widehat{\sigma} = \sigma \widehat{g}_N$ with $(1 + \sigma)\sigma = 2W$.

In the presented calculations we assume that the quality of both surfaces of the cavity is approximately equal and thus characterised by the same specularity parameter S, as is true

with silicon-silicon sample containers having even ^{4}He preplating between the surfaces. Example energy gap profiles corresponding to different specularities are shown in Supplementary Fig. 7 and density of states calculations in Fig. 3c in the main text.

SUPPLEMENTARY NOTE 4: Strong-coupling corrections

All calculations of energy gap Δ_A in the main text are based on quasiclassical weak-coupling theory adjusted for strong-coupling effects near T_c0. This is straightforward since the energy gap in the G-L regime is written as [2]

$$\Delta_\mathrm{A}^2 = \frac{\Delta C_\mathrm{A}}{C_\mathrm{N}} \left(\pi k_\mathrm{B} T_\mathrm{c0}\right)^2 \left(1 - T/T_\mathrm{c0}\right), \tag{18}$$

where ΔC_A refers to change in the specific heat at superfluid transition and C_N is the normal state specific heat. From this the initial slope of the gap acquires a simple form (see also Supplementary Eq. (6)):

$$\mathrm{IS}_\Delta^\mathrm{bulk} = \frac{\Delta C_\mathrm{A}}{C_\mathrm{N}} \left(\pi k_\mathrm{B} T_\mathrm{c0}\right)^2. \tag{19}$$

Thus, the ratio between the reported pressure-dependent value of the specific-heat jump $\frac{\Delta C_\mathrm{A}}{C_\mathrm{N}}$ [21] and its weak-coupling value 1.188 can be used as a pressure-dependent correction factor for both weak-coupling Δ_A^2 and $\mathrm{IS}_\Delta^\mathrm{bulk}$. We assume that Supplementary Eq. (18) is also valid for non-bulk values of the gap, $\left\langle \Delta_\mathrm{A}^2(z) \right\rangle$, with suppressed superfluid transition temperature T_c to make a similar trivial correction for non-specular boundary conditions, see Fig. 2c in the main text.

However, the strong-coupling effects in general depend both on temperature as well as on pressure. For ^{3}He-B these effects have been included in the weak-coupling-plus model [11]. The corresponding calculations [22] extended for thermodynamic properties of bulk ^{3}He-A (such as the maximum energy gap Δ_A) show very good agreement with our measurements under "specular" boundary conditions, see Supplementary Fig. 8. These calculations assume the gap to be uniform, i.e., do not take into account the nodes in the gap structure of ^{3}He-A, but it is to be expected that the inclusion of the nodal excitations will further improve the agreement. Similar calculations for different surface scattering boundary conditions do not yet exist, but it is our assessment that the low-temperature deviation between our "diffuse" measurement and the trivially corrected weak-coupling $S = 0.10$ calculation visible at 5.50 bar in Fig. 2c in the main text and in Supplementary Fig. 9 evidences the need for the same kind of temperature-dependent strong-coupling corrections when the average gap is suppressed by confinement.

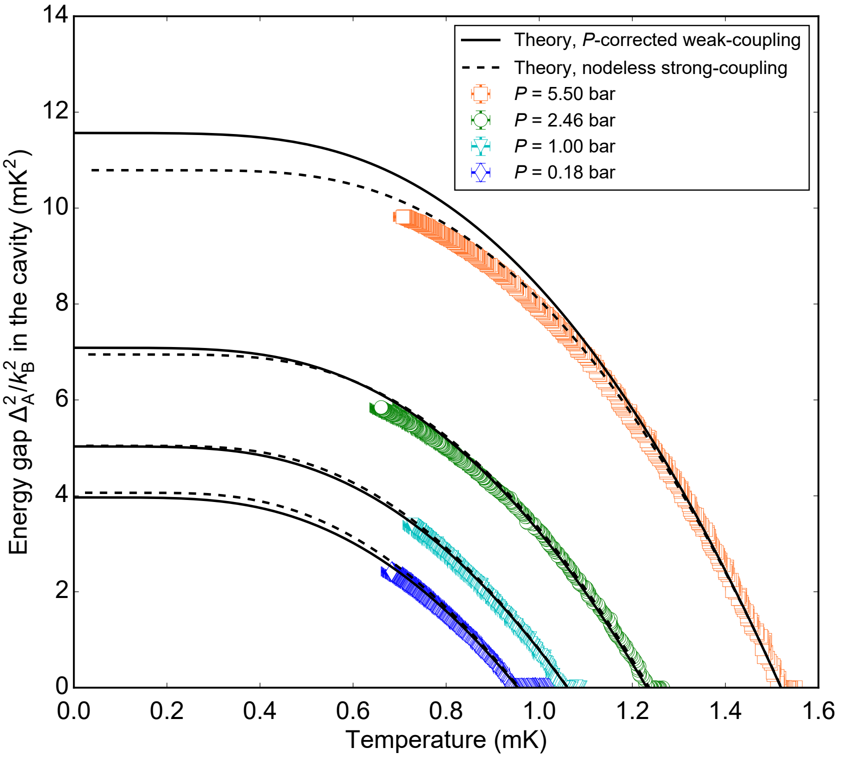

Supplementary Figure 8: Strong-coupling corrections to bulk energy gap. Measured energy gap in the cavity (symbols) under "specular" boundary condition agrees well with calculated bulk energy gap Δ_A including temperature-dependent strong-coupling corrections (dashed lines), see text. The result neglecting the temperature dependence of strong-coupling corrections, also shown in Fig. 2c in the main text, is plotted here for comparison (solid lines). Horizontal error bars show the uncertainty in temperature determination (Supplementary Note 7), and small vertical error bars show the uncertainty in the conversion between frequency shift and energy gap, arising from the uncertainty in the measured initial slope $\mathrm{IS}_{\mathrm{slab}}^{\prime\mathrm{spec}\prime}$ (Supplementary Eq. (7)).

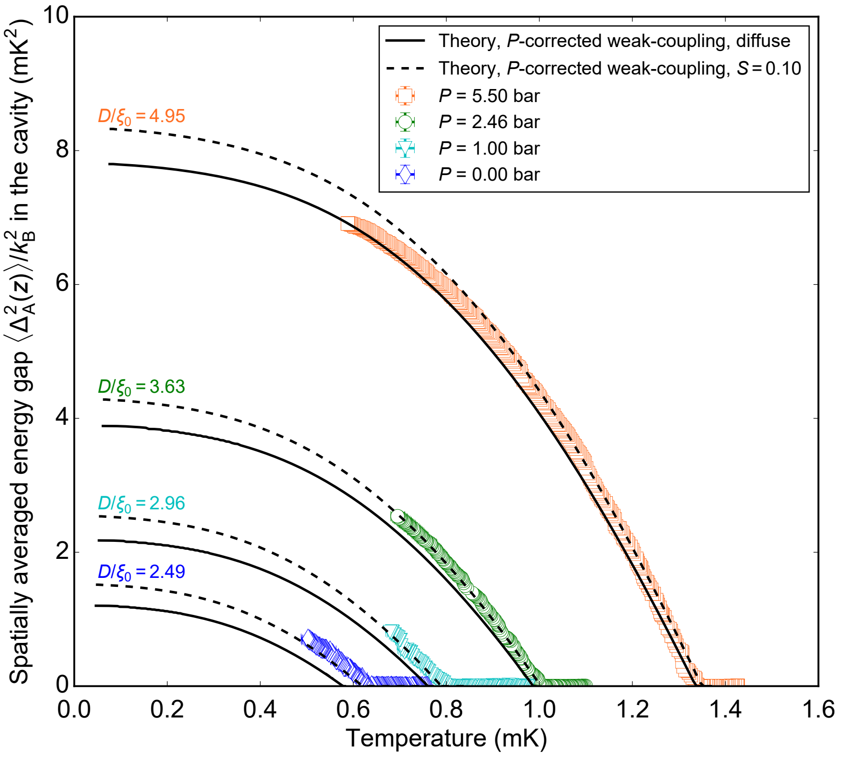

Supplementary Figure 9: Suppression of energy gap with "diffuse" boundary condition. Measured spatially averaged energy gap in the cavity with walls preplated with solid layer of ^{4}He (symbols) agrees best with theory for specularity $S = 0.10$. This is most evident at the three lowest pressures where no deviation from the weak-coupling temperature dependence is detected within our experimental temperature range. At 5.50 bar the low-temperature deviation from the theory is expected to arise from the strong-coupling effects in a similar way to the "specular" boundary condition in Supplementary Fig. 8. Here the theoretical curves for both the fully diffuse boundary condition $S = 0.00$ (solid lines) and specularity $S = 0.10$ (dashed lines) correspond to mean value of effective cavity height D/ξ_0 at each reported pressure, taking into account the uncertainty in cavity height measurement and the weak height distortion by pressure (Supplementary Fig. 2).

SUPPLEMENTARY NOTE 5: Determination of T_c in presence of solid ^{3}He surface boundary layer

With pure ^{3}He in the sample container, localised ^{3}He atoms next to a wall form a magnetic surface boundary layer [23, 24]. The susceptibility of this layer has been shown to obey Curie-Weiss law $\chi_s = C/(T-\theta)$, where C is the Curie constant, at high temperatures $T \gtrsim 1\,\mathrm{mK}$ with positive Weiss temperature θ, characteristic for systems with a ferromagnetic tendency [25]. The rest of the cavity is filled by liquid having a normal state ^{3}He susceptibility χ_N, whether in normal or in superfluid ^{3}He-A state. Total magnetisation of the sample is written as $M = M_s + M_\mathrm{FL}$, where $M_s \propto \chi_s$ represents the magnetisation of the solid layer and $M_\mathrm{FL} \propto \chi_\mathrm{N}$ is the liquid's magnetisation. The Fermi liquid susceptibility is well described by the phenomenological expression given by Dyugaev, $\chi_\mathrm{N} \propto 1/\sqrt{T^2 + T_\mathrm{F}^{**2}}$ [26], where effective Fermi temperature T_F^{**} is a density-dependent parameter [27–29]. In our experimental temperature range $T \ll T_\mathrm{F}^{**} \approx 200 - 300\,\mathrm{mK}$, the liquid magnetisation is constant, thus reducing the temperature dependence of M to result purely from the solid layer.

Temperature dependence of total magnetisation M in the cavity at two pressures is shown in Supplementary Fig. 10a. Magnetisation is determined from Lorentzian fits to Fourier-transformed data. Unlike in Refs. [30, 31], we do not observe line broadening as a function of temperature possibly due to extreme smoothness of the silicon surfaces. The constant magnetisation of the liquid is measured independently when having $32\,\mu\mathrm{mol\cdot m^{-2}}$ of ^{4}He in the sample, i.e., "diffuse" boundary condition and no evidence of temperature-dependent magnetisation since ^{4}He atoms have replaced all the localised ^{3}He on the walls.

Plotting the inverse of solid magnetisation against temperature, as shown in Supplementary Fig. 10b, lets us determine the Weiss temperature as the intersection between the linear fit and $1/M_s = 0$. This gives θ between 0.65 and 0.75 mK, which is consistent with earlier reported values changing between 0.3 and 0.8 mK [23, 25, 30, 32]. Possible systematic error follows from the shape of frequency spectra at $T \gtrsim 0.9T_{c0}$ where the spectral closeness of the signals arising from the bulk markers distorts the shape of the cavity signal, thus increasing the uncertainties in Lorentzian fitting.

We use the ferromagnetic high-temperature series expansion (HTSE) for triangular lattice up to 9th order in J_χ/T to fit the low-temperature values of M_s to be used in further analysis, Supplementary Fig. 10a. The exchange coefficient is defined as $J_\chi = \theta/3$. We have used the series expansion coefficients corresponding to Heisenberg model taking into account only two-particle exchange as given in Ref. [33]. These agree with the coefficients found for cyclic multiple-spin-exchange (MSE) model when only the dominant two and three-particle exchange processes (the effective Heisenberg exchange) are considered [34]. HTSE has earlier been used to successfully model solid ^{3}He layer on graphite [24, 31, 35, 36]. To avoid problems due to uncertain values of M near and above the bulk superfluid transition temperature T_{c0}, only the values extracted at temperatures $T \lesssim 0.9T_{c0}$ are used in fitting. This way we find $J_\chi \approx 0.15\,\mathrm{mK}$ which corresponds to the reported values of θ.

Due to the two-dimensional nature of the magnetic ^{3}He surface boundary layer, the local field of the oriented neighbouring spins results in negative frequency shift $\Delta f_s \propto M_s$ in NMR measurements when $\mathbf{H}_0 = H_0\hat{\mathbf{z}}$ is oriented normal to the surface [25, 30]. The atomic exchange between the solid and liquid components results in a single peak in the spectrum. The amplitude, frequency, and line shape of this composite signal depend on various factors, such as the intrinsic magnetisations and relaxation rates of the solid and the liquid as well as their exchange rates and the relative frequency shift [25]. The detected frequency shift of the composite peak, Δf, is determined as a weighted average of the intrinsic frequency shifts Δf_s and Δf_sf (superfluid) [38]:

$$\Delta f = \frac{M_s}{M}\Delta f_s + \frac{M_\mathrm{FL}}{M}\Delta f_\mathrm{sf}. \tag{20}$$

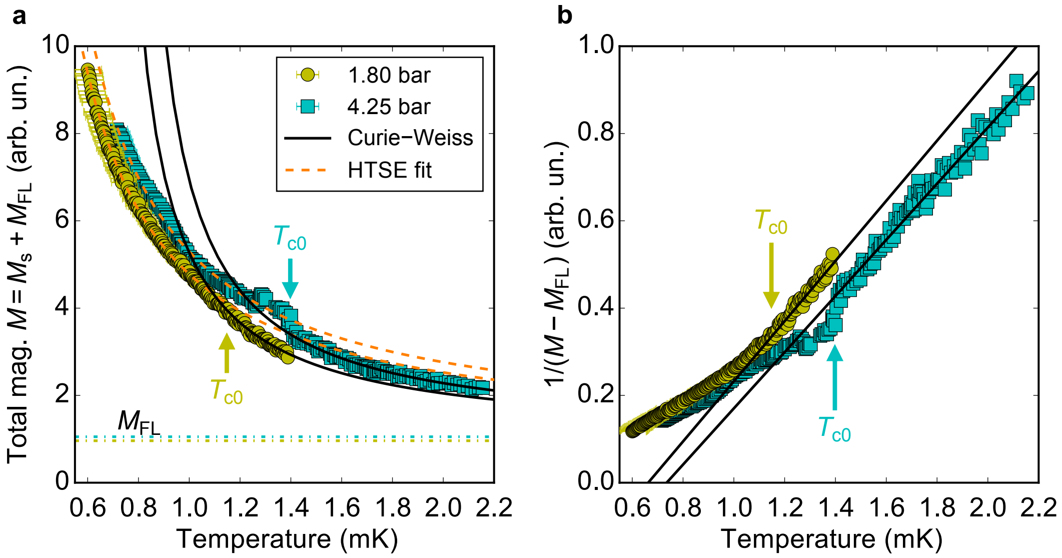

Supplementary Figure 10: Magnetisation with solid layer of ^{3}He on the cavity walls. **a** Total magnetisation M versus temperature at two different pressures (symbols). Independently determined liquid magnetisations $M_{\rm FL}$ are shown as horizontal dash-dotted lines and bulk superfluid transition temperatures $T_{\rm c0}$ are marked with arrows. At temperatures $T \gtrsim 1\,$mK the magnetisations are seen to follow the Curie-Weiss law (solid lines) and at temperatures $T \lesssim 0.9T_{\rm c0}$ the data are fit using HTSE (dashed lines, the deviations from these fits at higher temperatures are due to systematic errors in determination of M). Small pressure dependence of M on top of the almost constant $M_{\rm FL}$ results from the changing solid magnetisation $M_{\rm s}$ due to the compression of the solid layer with increasing pressure [27, 32, 37]. **b** The Weiss temperature θ is defined as the abscissa of the linear fit (solid lines) to the $1/M_{\rm s}$ versus temperature at $1/M_{\rm s} = 0$. The visible kink just below $T_{\rm c0}$, especially seen in 4.25 bar data, results from Lorentzian fitting of three close peaks (cavity signal and two bulk marker signals) being unreliable in determining the magnetisations of the individual features. Horizontal error bars indicate the uncertainty in temperature determination (Supplementary Note 7).

In normal state we have $\Delta f_{\rm sf} = 0$ and in the superfluid state its sign and magnitude depend on the superfluid phase, whereas the solid-induced temperature-dependent shift $\Delta f_{\rm s}$ is present regardless of the liquid being normal or superfluid. In the nanofluidic cavity of our sample cell, the ratio of solid-to-liquid magnetisation is high, $M_{\rm s} > M_{\rm FL}$. Thus, the solid frequency shift dominates the overall temperature dependence, masking the straightforward detection of superfluid transition in the cavity. Isolation of the signal arising from the solid is therefore a necessary prerequisite for the extraction of $T_{\rm c}$. See Supplementary Fig. 5 for measured frequency shifts corresponding to three different scattering boundary conditions.

We adopt two methods, with consistent results, to extract the superfluid $T_{\rm c}$ in the cavity in the presence of high background frequency shift arising from the solid ^{3}He:

(1) Direct comparison of frequency shifts measured at different pressures. At zero pressure the superfluidity is completely suppressed down to the lowest temperatures investigated. Thus, the zero-pressure frequency shift is well-described by HTSE fit over the full temperature range, and the superfluid transition at any higher pressure is identified as the temperature where the

frequency shift deviates from this zero-pressure fit. This method is presented in Supplementary Fig. 11a,b and relies on the assumption that the solid magnetisation, and thus the resulting frequency shift, is not dependent on pressure.
(2) Extraction of superfluid frequency shift by removing the solid effect. First, we write the solid frequency shift as $\Delta f_{\rm s} = C_{\rm s}M_{\rm s}$, where $C_{\rm s}$ is a proportionality constant. Now we get a two-domain frequency shift by using Supplementary Eq. (20):

$$\Delta f = C_{\rm s}\frac{(M - M_{\rm FL})^2}{M}, \text{ when } T > T_{\rm c}, \tag{21}$$

$$\Delta f = C_{\rm s}\frac{(M - M_{\rm FL})^2}{M} + \frac{M_{\rm FL}}{M}\Delta f_{\rm sf}, \text{ when } T < T_{\rm c}. \tag{22}$$

Thus, the frequency shift in the superfluid alone is

$$\Delta f_{\rm sf} = \frac{M}{M_{\rm FL}}\left(\Delta f - C_{\rm s}\frac{(M - M_{\rm FL})^2}{M}\right). \tag{23}$$

We determine $C_{\rm s}$ from a linear fit to the the measured Δf against $(M - M_{\rm FL})^2/M$ in range $T \lesssim 0.9T_{\rm c0}$ to as low temperature as the data show linearity (at zero pressure to the lowest temperature investigated). This can be done individually for each data set (Supplementary Fig. 11c), or, if assuming pressure-independence, we can use the zero-bar $C_{\rm s}$ at every pressure (Supplementary Fig. 11d).

Both methods (1) and (2) extract a clear break in the temperature dependence of the frequency shift, making the determination of superfluid transition straightforward. The values of $T_{\rm c}$ corresponding to solid ^{3}He boundary condition plotted in Fig. 3 in the main text are based on method (2), using individually determined $C_{\rm s}$. However, whichever method we use, the values coincide with each other. Due to uncertainties in determination of $M_{\rm s}$ and $C_{\rm s}$, we do not consider the extracted temperature dependence of the superfluid frequency shift (Supplementary Fig. 11c,d) to be reliable. A significant improvement is possible by conducting measurements in lower magnetic fields, which both increases the absolute value of $\Delta f_{\rm sf}$ and reduces the absolute magnitude of the dipolar frequency shift $\Delta f_{\rm s}$ arising from the solid.

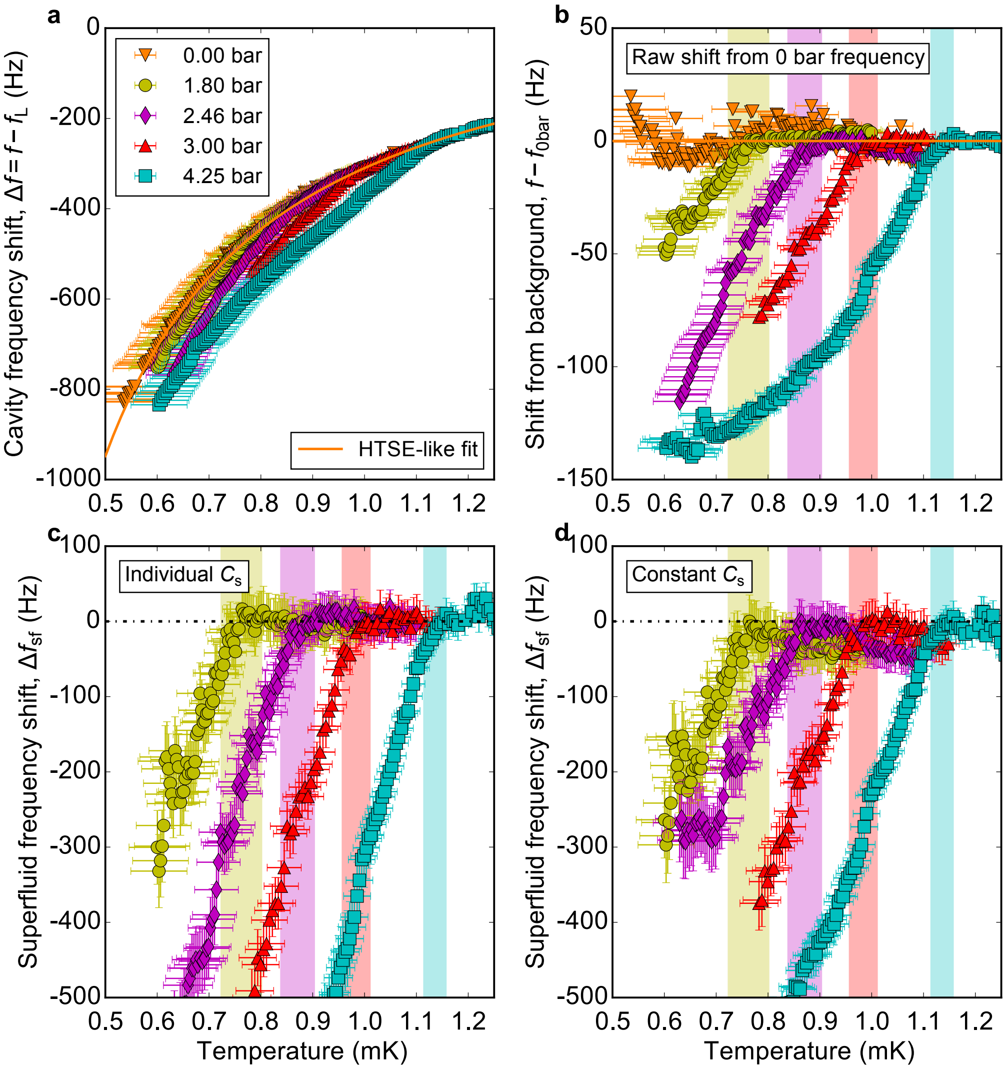

Supplementary Figure 11: Superfluid frequency shift in the cavity with solid ^{3}He on the walls. a Measured NMR frequency shifts of the cavity signal (symbols) at higher pressures deviate from the low-pressure values at certain temperatures, indicating a superfluid transition in the liquid (method (1)). Solid line is a fit to 0 bar data using high-temperature series expansion (HTSE) as a model for the solid magnetisation M_s. To compensate the small pressure dependence in M_s, the Larmor frequency $f_L \approx 967\,$kHz of each dataset is adjusted by less than 20 Hz to make Δf agree with the 0 bar HTSE fit at $T = 0.9T_{c0}$ of each pressure. **b** The temperatures at which the deviations occur are clearly detected when the “background” frequency shift at 0 bar is subtracted from the data. **c**–**d** The pure superfluid frequency shifts Δf_{sf} without the solid contribution can be extracted using method (2) with individually determined proportionality constant C_s or with constant zero-bar C_s as described in the text. The latter way clearly gives smaller Δf_{sf}. The coloured vertical bands in **b**, **c**, and **d** indicate the values of superfluid transition temperature in the cavity, T_c, with uncertainty, determined using the method illustrated in **c**. It is seen that all the techniques showcased here give a consistent result. Horizontal error bars indicate the uncertainty in temperature determination (Supplementary Note 7) and vertical error bars in **c** and **d** reflect the uncertainty in the proportionality constant C_s.

SUPPLEMENTARY NOTE 6: Pair breaking at the surface

Momentum scattering. The scattering of quasiparticles from surface and the consequential pair breaking are incorporated into the quasiclassical theory in a form of boundary conditions for the propagator (see also Supplementary Note 3). An efficient way to write them is to connect the coherence amplitude $\Gamma(\mathbf{p}_{\rm out})i\sigma_y$ on the outgoing trajectory with the incoming amplitudes $\gamma(\mathbf{p}_{\rm in})i\sigma_y$ through scattering matrix [39],

$$\Gamma(\mathbf{p}_{\rm out})i\sigma_y = \mathcal{S}\ [\gamma(\mathbf{p}_{\rm in})i\sigma_y]\ \tilde{\mathcal{S}}, \tag{24}$$

where $\mathcal{S}$ is the normal state scattering matrix in the particle sector and $\tilde{\mathcal{S}}$ in the hole sector. Coherence amplitudes carry the information about the structure of the order parameter, and the difference between the asymptotic value $\gamma(\mathbf{p}_{\rm out})$ and the initial scattered value $\Gamma(\mathbf{p}_{\rm out})$ of coherence amplitude on the outgoing trajectory can give an indication of the pair-breaking properties of the surface. Far from the surface, the asymptotic value on any trajectory $\mathbf{k}$ is

$$\gamma(\mathbf{k}) \equiv \gamma_0 + \boldsymbol{\gamma}_{\rm t}(\mathbf{k})\cdot\boldsymbol{\sigma} = \gamma_0 + \gamma_x\sigma_x + \gamma_y\sigma_y + \gamma_z\sigma_z. \tag{25}$$

Here γ_0 and $\boldsymbol{\gamma}_{\rm t}(\mathbf{k}) = \gamma_x(\mathbf{k})\hat{\mathbf{x}} + \gamma_y(\mathbf{k})\hat{\mathbf{y}} + \gamma_z(\mathbf{k})\hat{\mathbf{z}}$ are the singlet and triplet components in spin space, respectively, and $\boldsymbol{\sigma} = \sigma_x\hat{\mathbf{x}} + \sigma_y\hat{\mathbf{y}} + \sigma_z\hat{\mathbf{z}}$ is the Pauli vector consisting of Pauli matrices σ_x, σ_y, and σ_z. For unitary phases coherence amplitudes reflect symmetry of order parameter $\Delta(\mathbf{k})$ [15]: $\gamma_0 i\sigma_y \propto \Delta_0 i\sigma_y$ and $(\boldsymbol{\gamma}_{\rm t}(\mathbf{k})\cdot\boldsymbol{\sigma})\,i\sigma_y \propto \Delta_{\rm t}(\mathbf{k}) = (\mathbf{d}(\mathbf{k})\cdot\boldsymbol{\sigma})\,i\sigma_y$, where $\mathbf{d}(\mathbf{k})$ is the order-parameter vector.

Now we consider ^{3}He-A (spin-triplet pairing) with $\mathbf{d}(\mathbf{k}) \perp \mathbf{H}_0 = H_0\hat{\mathbf{z}}$ relevant to our experiments [2]:

$$\mathbf{d}(\mathbf{k}) = \hat{\mathbf{x}}\Delta_{\rm A}(\hat{k}_x + i\hat{k}_y) \quad \propto \quad |\uparrow\uparrow\rangle + |\downarrow\downarrow\rangle. \tag{26}$$

For the purely momentum scattering one can employ the surface roughness averaging over incoming directions that leads to

$$\Gamma(\mathbf{p}_{\rm out}) = |S|\gamma(\mathbf{p}_{\rm in}) + (1-|S|)\langle\gamma\rangle_{\parallel} = |S|\gamma(\mathbf{p}_{\rm in}), \tag{27}$$

where the coherence parameter $0 \le |S| \le 1$, as defined below Supplementary Eq. (13), describes the relative amount of quasiparticles scattering coherently (non-diffusively) between $\mathbf{p}_{\rm in}$ and $\mathbf{p}_{\rm out}$, and for A phase the momentum average over all in-plane directions (diffuse scattering) gives zero, $\langle\gamma\rangle_{\parallel} \propto \langle p_x + ip_y\rangle_{\parallel} = 0$.

The scattering is associated with a relative phase change ϕ on the order parameter "seen" by the quasiparticle on the incoming and the outgoing trajectories: for skew scattering in the A phase $\Delta(\mathbf{p}_{\rm out}) = e^{-i\phi}\Delta(\mathbf{p}_{\rm in})$. As shown in Ref. [15], this scattering phase results in the energy spectrum of surface-bound states: $E_{\rm SBS}/\Delta_{\rm A} = \pm\cos\phi/2$. For purely momentum scattering in ^{3}He-A, ϕ corresponds to x-y plane rotation in momentum space. Fully specular scattering ($|S| = 1, \phi = 0$) generates no pair breaking and no additional sub-gap energy states at the surface, giving the relation $\Gamma(\mathbf{p}_{\rm out}) = \gamma(\mathbf{p}_{\rm in}) = \gamma(\mathbf{p}_{\rm out})$ for the coherence amplitudes. The completely backscattering (retroreflective) surface $\mathbf{p}_{\rm out} = -\mathbf{p}_{\rm in}$ with $|S| = 1$ and $\phi = \pi$ leads to largest weight of zero-energy surface states, $E_{\rm SBS} = 0$, and gives $\Delta(\mathbf{p}_{\rm out}) = -\Delta(\mathbf{p}_{\rm in})$ on all scattering trajectories, fulfilling the condition of coherence amplitudes generating maximal pair breaking and suppression of $T_{\rm c}$: $\Gamma(\mathbf{p}_{\rm out}) = \gamma(\mathbf{p}_{\rm in}) = -\gamma(\mathbf{p}_{\rm out})$. In purely diffuse case ($|S| = 0$) the scattering angle ϕ is fully diffuse, i.e., evenly spread over the entire phase circle between $\phi = 0$ and $\phi = 2\pi$, resulting in all the possible bound state energies filling up the gap uniformly. Partially diffuse scattering has $0 < |S| < 1$. All this is visible in the calculations of DOS, Fig. 3c in the main text.

In general, the quasiparticle reflection picks up relative phase ϕ, responsible for bound-state energies, from both momentum and magnetic degrees of freedom between the incoming and outgoing trajectories. We investigate below whether the spin dependence in scattering can increase the density of surface-bound states close to zero energy ($\phi = \pi$) and thus result in more than diffuse pair breaking even in the absence of partial retroreflection by the surface. For the A phase with scattering path $\gamma(\mathbf{p}_{\rm out}) = \gamma(\mathbf{p}_{\rm in})$, maximal pair breaking will occur when $\Gamma(\mathbf{p}_{\rm out}) = -1 \cdot \gamma(\mathbf{p}_{\rm in})$.

Magnetic scattering from polarized surface. The magnetically active highly-polarizable solid layer of ^{3}He on the surface can affect the spin structure of the superfluid phase stabilised in the cavity. These effects will originate from the spin-dependent scattering of the quasiparticles from the solid layer and should be included in the boundary condition for the quasiclassical propagator. On the general grounds one expects that such effects could be magnetically anisotropic and strongly depend on the spin structure of the superfluid phase and the orientation of the magnetic field. Here we are particularly interested in the role of magnetic scattering on the suppression of $T_{\rm c}$.

Magnetic scattering in this formalism is included through the spin-dependent part $\mathcal{M}$ of the $\mathcal{S}$-matrix. This matrix has the form [40]

$$\mathcal{M} = \begin{pmatrix} e^{-i\delta_{\uparrow\mathbf{m}}} & \\ & e^{-i\delta_{\downarrow\mathbf{m}}} \end{pmatrix} = e^{-i\vartheta_0} e^{-i(\hat{\mathbf{m}}\cdot\boldsymbol{\sigma})\vartheta/2} \tag{28}$$

and it describes the phase difference $\vartheta = \delta_{\uparrow\mathbf{m}} - \delta_{\downarrow\mathbf{m}}$ that spins up and down (in $\hat{\mathbf{m}}$-basis) acquire when they scatter off the (classically) magnetically-polarized surface. Here unit vector $\hat{\mathbf{m}}$ refers to the polarization axis of magnetisation of the surface layer. The hole sector matrix is obtained assuming particle-hole symmetry $\tilde{\mathcal{M}} = \mathcal{M}^*$. The spin-dependent phase difference can be related, for example, to the exchange coupling in the ferromagnetic layer [41], or one can consider a possibility of Kondo-like resonant scattering that may enhance the effective exchange interaction and produce large phase shifts [42]. However, in general the phase shift can be treated as a model-dependent parameter.

In a basic model, where the effects of momentum and spin rotation during scattering event can be thought to be independent of each other, the combined form of the coherence amplitude after scattering is

$$\begin{aligned}\Gamma(\mathbf{p}_{\rm out}) i\sigma_y = |S|\mathcal{M}\left[\gamma(\mathbf{p}_{\rm in}) i\sigma_y\right]\tilde{\mathcal{M}} &= |S| e^{-i(\hat{\mathbf{m}}\cdot\boldsymbol{\sigma})\vartheta/2}(\gamma_0 + \boldsymbol{\gamma}_{\rm t}(\mathbf{p}_{\rm in})\cdot\boldsymbol{\sigma}) e^{-i(\hat{\mathbf{m}}\cdot\boldsymbol{\sigma})\vartheta/2}(i\sigma_y) \\ &= \left[|S| e^{-i\sigma_z\vartheta}(\gamma_0 + \gamma_z\sigma_z) + |S|(\gamma_x\sigma_x + \gamma_y\sigma_y)\right](i\sigma_y),\end{aligned} \tag{29}$$

where in the last step it has been taken into account that the direction of magnetisation in the solid layer is along the external field, $\hat{\mathbf{m}} \parallel \hat{\mathbf{z}}$. One notes that, first, due to decoupled spin and momentum spaces only the coherent part of momentum reflection from Supplementary Eq. (27) contributes, since the diffuse part of scattering averages the order parameter to zero and magnetically-induced phase shifts are not affecting this average. Second, the γ_x and γ_y components of triplet $\boldsymbol{\gamma}_{\rm t}$ are not affected by magnetic scattering, meaning that $|\uparrow\uparrow\rangle$ and $|\downarrow\downarrow\rangle$ pairs ($S_{\mathbf{m}} = \pm 1$) are not magnetically suppressed, since the spins of these pairs scatter with the same phase. Thus, in geometry such as ours, magnetic scattering of this type does not suppress $T_{\rm c}$ of ^{3}He-A further from the value set by momentum scattering.

For magnetic scattering to have effect, the pairs must be in $S_{\mathbf{m}} = 0$ state, where spins of the $|\uparrow\downarrow + \downarrow\uparrow\rangle$ pairs would scatter with different phases. The total phase accumulated by the quasiparticle during the scattering, and thus the energy of surface-bound state, is a combination of rotation of γ_z component in spin space and the difference in $\Delta(\mathbf{k})$ due to scattering in momentum space. These two can either enhance or cancel each other's effect on pair breaking. Assuming no pair breaking due to momentum scattering, $S_{\mathbf{m}} = 0$ pairs would be completely broken when the γ_z component is rotated by $\vartheta = \pi$ generating zero-energy surface-bound states, which would be the case of resonant magnetic scattering.

The mixing of all pairs ($S_{\mathbf{m}} = 0, \pm 1$) in our experimental configuration becomes possible if the direction of magnetization in the solid layer is random. The average over $\hat{\mathbf{m}}$-angles gives partial suppression of all amplitudes at the surface:

$$\Gamma(\mathbf{p}_{\rm out}) i\sigma_y = |S| \int \frac{d\Omega_{\hat{\mathbf{m}}}}{4\pi} \mathcal{M}(\hat{\mathbf{m}}) \left[\gamma(\mathbf{p}_{\rm in}) i\sigma_y\right] \tilde{\mathcal{M}}(\hat{\mathbf{m}})$$
$$= \left[|S|\gamma_0 \cos\vartheta + |S| (\boldsymbol{\gamma}_{\rm t}(\mathbf{p}_{\rm in}) \cdot \boldsymbol{\sigma}) \left(1 - \frac{2}{3}\sin^2\frac{\vartheta}{2}\right)\right] (i\sigma_y). \tag{30}$$

In this case the magnetic suppression cannot take the total suppression beyond diffuse due to non-negative coefficient for the triplet components.

The only scenario increasing pair breaking enough in this model in order to get more than diffuse suppression of $T_{\rm c}$ in ^{3}He-A, is to allow for correlated spin-orbital scattering, where backscattering quasiparticles experience phase difference due to orbital part of the order parameter alone, and the forward-scattering quasiparticles are affected by magnetic depairing. The latter part would also require a certain orientation of magnetization in the solid layer, inconsistent with the experimental geometry. From the physical angle, this scenario also appears to be unlikely due to absence of plausible spin-orbital coupling mechanism in the layer.

Another way to formulate the boundary condition for the coherence amplitudes is to model the magnetic layer by a net of polarized or unpolarized scattering centres with potential and exchange interaction $u_0 + J\mathbf{S}_{\rm imp} \cdot \boldsymbol{\sigma}$ with a classical magnetic moment $\mathbf{S}_{\rm imp}$ of the centres ("impurities") and liquid-solid exchange coupling coefficient J. These act to randomize the directions of scattered quasiparticles, but introduce in general different relaxation times for quasiparticles of different spins. In this modification of Ovchinnikov-Kopnin model of thin dirty layer [43, 44] with thickness d, one again encounters vanishing diffuse average over directions $\langle\gamma\rangle_{\parallel} = 0$ that does not further contribute to the magnetic pair breaking. The coherent scattering part is given by spin-dependent relaxation lengths ℓ, e.g., $\Gamma(\mathbf{p}_{\rm out}) = \gamma(\mathbf{p}_{\rm in}) e^{-2d/\ell}$ with $v_{\rm F}/\ell_{\pm} = 2\pi n_{\rm imp} N_{\rm F} (u_0 \pm JS_{\rm imp})^2$ for $S_z = \pm 1$ pairs, or $v_{\rm F}/\ell_0 = 2\pi n_{\rm imp} N_{\rm F} (u_0^2 + (JS_{\rm imp})^2)$ for $S_z = 0$ pairs, where $n_{\rm imp}$ is the density of scattering centres in the magnetic layer. These expressions are consistent with previous work on bulk systems in aerogel, and they may lead to effects such as A_1-A_2 phase splitting [45], but will not result in an excess of zero-energy states because $e^{-2d/\ell}$ coefficients are all positive. The maximal allowed pair breaking is diffuse, $|S| = 0$, in the limit $d \gg \ell$ with no magnetic effects discernible.

Quantum spin scattering. We propose to extend the boundary conditions to take into account the quantum interference of spin states of the (identical) solid and liquid ^{3}He atoms. They will include spin-flip processes and more subtle scattering effects that are missing in the classical magnetic moment approaches. A rough model for the scattering of a liquid ^{3}He quasiparticle in the solid layer of thickness a is given by the Hamiltonian in the solid layer

$$\mathcal{H} = -\frac{\hbar^2}{2m}\boldsymbol{\nabla}^2 + U_0 + J\hat{\mathbf{S}}^s \cdot \frac{\hbar}{2}\boldsymbol{\sigma}, \tag{31}$$

where U_0 is the potential height of the barrier, $\hat{\mathbf{S}}^s$ is the $\hbar/2$ spin operator of solid layer atom, and $\frac{\hbar}{2}\boldsymbol{\sigma}$ is the spin operator of liquid quasiparticle. Below the layer, we set up an impenetrable wall so that all particles are reflected back. The total spin of the quasiparticle and the solid atom participating in scattering can be in the singlet or triplet state, $S_{\rm tot} = 0, 1$, with the standard eigenvalues for the product $\langle \hat{\mathbf{S}}^s \cdot \frac{\hbar}{2}\boldsymbol{\sigma}\rangle = -\frac{3}{4}\hbar^2, +\frac{1}{4}\hbar^2$. The scattering matrix $e^{-ikz} \to -e^{i\alpha}e^{ikz}$ is (omitting overall -1 factor)

$$\mathcal{M} = \begin{bmatrix} e^{i\alpha_t} & & & \\ & e^{i\alpha_s} & & \\ & & e^{i\alpha_t} & \\ & & & e^{i\alpha_t} \end{bmatrix} \quad \text{in total spin basis} \quad \begin{pmatrix} S_{\rm tot} = 1, & m = +1 \\ S_{\rm tot} = 0, & m = \ \ 0 \\ S_{\rm tot} = 1, & m = \ \ 0 \\ S_{\rm tot} = 1, & m = -1 \end{pmatrix}, \tag{32}$$

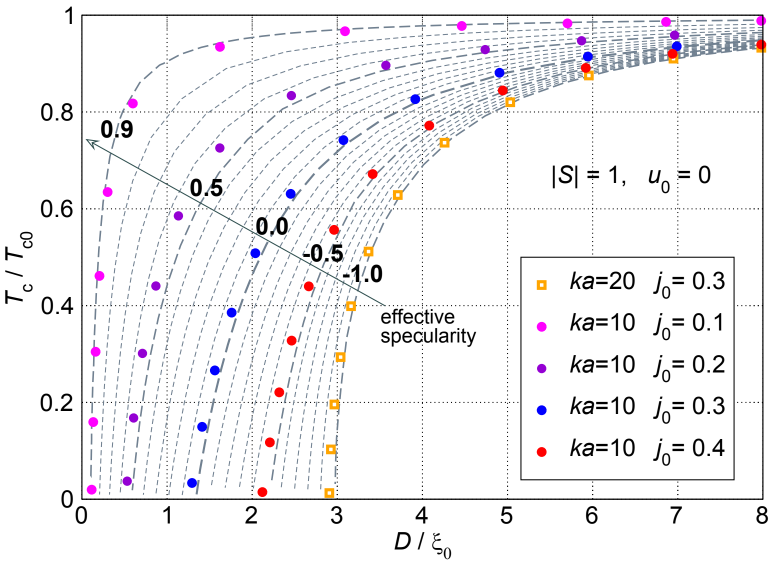

Supplementary Figure 12: Suppression of $T_{\rm c}$ by magnetic scattering on quantum spins. The momentum scattering is taken to be fully specular, $|S| = 1$, reducing the suppression to be purely from magnetic origin. Dashed lines show the calculated suppression of $T_{\rm c}$ corresponding to different effective specularities $-1 \le S_{\rm eff} \le 1$. Magnetic scattering, Supplementary Eq. (40), is parametrized by four scattering phases, $\alpha_{s,t}, \tilde{\alpha}_{s,t}$, in singlet and triplet channels for both particles and holes. The values for these depend on microscopic model of the magnetic layer. Symbols show the resulting suppression corresponding to a set of chosen layer parameters, assuming the orders of magnitude $k \sim k_{\rm F} \sim 10\,{\rm nm}^{-1}$ and $a \sim 1\,{\rm nm}$ and no scalar potential in the layer, $u_0 = 0$. We also neglect the dependence of the scattering phases on momentum perpendicular to the wall. Adjusting the parameters, we can model the suppression over the full range of effective specularity.

where the phases of the singlet and triplet scattering channels are given by

$$\tan\frac{\alpha_s}{2} = \frac{\tan\left(ka\sqrt{1-u_0+\frac{3}{4}j_0}\right)}{\sqrt{1-u_0+\frac{3}{4}j_0}}\,, \qquad \tan\frac{\alpha_t}{2} = \frac{\tan\left(ka\sqrt{1-u_0-\frac{1}{4}j_0}\right)}{\sqrt{1-u_0-\frac{1}{4}j_0}} \tag{33}$$

with definitions

$$u_0 = \frac{2mU_0}{\hbar^2 k^2}\,, \qquad j_0 = \frac{2mJ}{k^2}\,. \tag{34}$$

We transform to the basis labelled by z-projections of liquid (σ, σ') and solid $(S_z^s, S_z^{s'})$ spins before and after the scattering event using Clebsch-Gordan matrix $\mathcal{C}$,

$$|\sigma, S_z^s\rangle = \begin{pmatrix} +1/2, +\frac{1}{2} \\ +1/2, -\frac{1}{2} \\ -1/2, +\frac{1}{2} \\ -1/2, -\frac{1}{2} \end{pmatrix} = \mathcal{C}^{-1} \begin{pmatrix} S_{\rm tot} = 1, & m = +1 \\ S_{\rm tot} = 0, & m = \ \ 0 \\ S_{\rm tot} = 1, & m = \ \ 0 \\ S_{\rm tot} = 1, & m = -1 \end{pmatrix}, \quad \mathcal{C} = \begin{bmatrix} 1 & & & \\ & \frac{1}{\sqrt{2}} & -\frac{1}{\sqrt{2}} & \\ & \frac{1}{\sqrt{2}} & \frac{1}{\sqrt{2}} & \\ & & & 1 \end{bmatrix}, \tag{35}$$

to get the scattering matrix

$$\mathcal{M}_{\sigma,\sigma'\otimes S_z^s,S_z^{s'}} = \mathcal{C}^{-1}\begin{bmatrix} e^{i\alpha_t} & & & \\ & e^{i\alpha_s} & & \\ & & e^{i\alpha_t} & \\ & & & e^{i\alpha_t}\end{bmatrix}\mathcal{C}$$
$$= e^{i\alpha_+/2}\left(\begin{array}{cc|cc} e^{-i\alpha_-/2} & & & \\ & \cos\frac{\alpha_-}{2} & -i\sin\frac{\alpha_-}{2} & \\ \hline & -i\sin\frac{\alpha_-}{2} & \cos\frac{\alpha_-}{2} & \\ & & & e^{-i\alpha_-/2}\end{array}\right)\Bigg|_{\alpha_\pm=\alpha_s\pm\alpha_t} . \quad (36)$$

The separate 2×2 blocks correspond to (σ,σ') space; within each block we have 2×2 $(S_z^s, S_z^{s'})$ space. One gets similar expression for the hole sector:

$$\tilde{\mathcal{M}}_{\sigma,\sigma'\otimes S_z^s,S_z^{s'}} = (-i\sigma_y)\mathcal{C}^{-1}\begin{bmatrix} e^{i\tilde{\alpha}_t} & & & \\ & e^{i\tilde{\alpha}_s} & & \\ & & e^{i\tilde{\alpha}_t} & \\ & & & e^{i\tilde{\alpha}_t}\end{bmatrix}\mathcal{C}(i\sigma_y) . \quad (37)$$

Here $(i\sigma_y)$-factors act on liquid spin variables, and the scattering phases

$$\tan\frac{\tilde{\alpha}_s}{2} = -\frac{\tan\left(ka\sqrt{1-u_0-\frac{3}{4}j_0}\right)}{\sqrt{1-u_0-\frac{3}{4}j_0}} , \qquad \tan\frac{\tilde{\alpha}_t}{2} = -\frac{\tan\left(ka\sqrt{1-u_0+\frac{1}{4}j_0}\right)}{\sqrt{1-u_0+\frac{1}{4}j_0}} \quad (38)$$

do not show the particle-hole symmetry, which was present in the scattering on a classical spin, due to difference between singlet and triplet factors $-3/4, +1/4$. (In large-spin or particle-hole-symmetric case, we would have $\tilde{\alpha}_s = -\alpha_t$, $\tilde{\alpha}_t = -\alpha_s$.)

By taking average of solid spin configurations, we obtain the boundary condition for the coherence amplitudes:

$$\Gamma_{\alpha\beta}(\mathbf{p}_{\rm out}) = \sum_{S_z^s=\pm\frac{1}{2}} \mathcal{P}(S_z^s)\,\langle S_z^s|\mathcal{M}_{\alpha,\sigma\otimes S_z^s,S_z^{s'}}\,\gamma_{\sigma\sigma'}(\mathbf{p}_{\rm in})\,\tilde{\mathcal{M}}_{\sigma',\beta\otimes S_z^{s'},S_z^s}|S_z^s\rangle . \quad (39)$$

In the assumption of unpolarized solid layer, $\mathcal{P}(+\frac{1}{2}) = \mathcal{P}(-\frac{1}{2}) = 1/2$ (i.e., the NMR field is not strong enough to orient significant fraction of the spins), the triplet components upon scattering from the magnetic layer are multiplied by an additional factor:

$$\begin{aligned}\Gamma_{x,y,z}(\mathbf{p}_{\rm out}) &= Q_{\rm sf}\,|S|\,\gamma_{x,y,z}(\mathbf{p}_{\rm in})\\ Q_{\rm sf} &= e^{i(\alpha_+ + \tilde{\alpha}_+)/2}\,\frac{1}{2}\left(e^{-i\alpha_-/2}\cos\frac{\tilde{\alpha}_-}{2} + e^{-i\tilde{\alpha}_-/2}\cos\frac{\alpha_-}{2}\right)\\ &= \frac{1}{4}\left(e^{i(\alpha_s+\tilde{\alpha}_t)} + e^{i(\tilde{\alpha}_s+\alpha_t)} + 2e^{i(\alpha_t+\tilde{\alpha}_t)}\right) .\end{aligned} \quad (40)$$

Complex magnetic pair-breaking parameter $Q_{\rm sf}$ is a mixture of singlet and triplet channel scattering phases of both particles and holes. It can easily become negative, depending on the specific values of the phases in Supplementary Eqs. (33) and (38) which in this model are functions of solid layer parameters ka and j_0 defined in Supplementary Eq. (34). We assume partially specular momentum scattering with less than diffuse $T_{\rm c}$ suppression. In this situation the largest increase in the density of zero-energy surface-bound states arises when $\alpha_s + \tilde{\alpha}_t \sim \pi$, $\tilde{\alpha}_s + \alpha_t \sim \pi$, and

$\alpha_t + \tilde{\alpha}_t \sim \pi$, so that the real part $\Re Q_{\rm sf} = (\cos(\alpha_s + \tilde{\alpha}_t) + \cos(\tilde{\alpha}_s + \alpha_t) + 2\cos(\alpha_t + \tilde{\alpha}_t))/4 \sim -1$. Further adjustment of the parameters can lead to total $T_{\rm c}$ suppression corresponding to any value of effective specularity $-|S| \leq S_{\rm eff} \leq |S|$. Thus, the suppression of $T_{\rm c}$ can be more than diffuse even for fully specular momentum scattering $|S| = 1$, in which case the magnetic pair breaking alone covers the full range of suppressions matching the effective specularity from specular to retroreflective (Supplementary Fig. 12).

In order to get $T_{\rm c}$ suppression matching effective specularity $S_{\rm eff} = -0.4$, as detected with solid ^{3}He surface boundary layer (see Fig. 3a in the main text), the underlying specularity of momentum scattering needs to be $S \geq 0.4$. This can in principle be possible if the atomically smooth silicon surfaces of the cavity [1] are not significantly roughened by solid ^{3}He layer, and if atomic-level roughness of the solid substrate itself can promote specular scattering.

The three mechanisms capable of producing $T_{\rm c}$ suppression more than diffuse, as discussed here, are all illustrated in Fig. 3b in the main text.

SUPPLEMENTARY NOTE 7: Temperature correction

We measure the temperature $T_{\rm Ag}$ of the silver plate on which the sample container sits. Due to thermal boundary resistance $R_{\rm K}$ across the sintered silver heat exchanger, there is a temperature gradient ΔT between the sample helium and the silver plate: $\Delta T = T_{\rm He} - T_{\rm Ag} = R_{\rm K}\dot{Q}$, where $T_{\rm He}$ is the helium temperature and $\dot{Q} = \dot{Q}_0 + \dot{Q}_{\rm NMR}$ is the heat flux into the sample [46]. Here residual constant heat flux is $\dot{Q}_0$ and the heat flux due to NMR pulses is $\dot{Q}_{\rm NMR}$. Typical form of boundary resistance is $R_{\rm K} = \frac{K}{T^\alpha}$, where K is a constant dependent on material and boundary properties [47]. At high temperatures we expect $\alpha = 3$, following from the acoustic mismatch of phonons at the interface. However, at low temperatures below 10 mK, α typically decreases towards unity and is different for pure ^{3}He and for mixtures of ^{4}He and ^{3}He [48, 49]. Therefore, we take α as a free parameter for each surface preplating investigated.

We fit α by measuring the change in ΔT as a function of $\dot{Q}$ across the heat exchanger over the whole temperature range of interest. This is done by performing full temperature sweeps using two different-sized NMR pulses denoted as pulse A and pulse B, where the total rf-field power generated by pulse A is ten times the power generated by pulse B. These pulses correspond to tipping angles $\beta \approx 10^\circ$ and $\beta \approx 3^\circ$, respectively. We denote the two significantly different NMR-induced heat fluxes into the sample by $\dot{Q}_{{\rm NMR},A}$ and $\dot{Q}_{{\rm NMR},B}$, which result in two different dependences of the superfluid frequency shift in the cavity on the measured silver-plate temperature (Supplementary Fig. 13a,c). Since any measured frequency shift Δf in the superfluid ^{3}He-A in the cavity, using small-tipping-angle pulses, always corresponds to the same helium temperature, we can calibrate the additional heating using equation

$$\int_{T_{\rm Ag}}^{T_{\rm He}} \frac{dT}{R_{\rm K}} = \frac{T_{\rm He}^{\alpha+1} - T_{\rm Ag}^{\alpha+1}}{(\alpha+1)K} = \dot{Q}. \tag{41}$$

From this it follows for any Δf

$$\dot{Q}_A - \dot{Q}_B = \dot{Q}_{{\rm NMR},A} - \dot{Q}_{{\rm NMR},B} = \dot{Q}_{{\rm NMR},A-B} = \frac{T_{{\rm Ag},B}^{\alpha+1} - T_{{\rm Ag},A}^{\alpha+1}}{(\alpha+1)K}. \tag{42}$$

Since K and $\dot{Q}_{{\rm NMR},A-B}$ are both constants, we can fit the measured temperature differences corresponding to each frequency shift to determine α. We find it to be independent of both temperature and pressure over the 0.5–1.5 mK, 0.0–5.5 bar range. With "specular" boundary condition $\alpha = 2.5$ and with "diffuse" $\alpha = 1.5$. These values collapse data of different pulses, see Supplementary Fig. 13a,c. We have also compared the frequency shifts using pulse B and a medium-sized pulse having four times the power generated by B. The frequency shifts match within the experimental error, leading us to conclude that the heating caused by pulse B is negligible, i.e., $\dot{Q}_{{\rm NMR},B} \approx 0$.

Given the determination of α for each boundary condition, we can make the final correction for residual heat leak into the sample, $\dot{Q}_0$. This is done by comparison of the measured silver plate temperature $T_{\rm Ag} \equiv T_{\rm c}^{\rm fill}$ during the superfluid transition in the fill line bulk marker, which is most directly connected to the heat exchanger, with the literature value given by Greywall, $T_{\rm He} \equiv T_{\rm c}^{\rm G}$ [10]. The comparison is made at each pressure for both pulse A and pulse B (Supplementary Fig. 13b,d). Referring to Supplementary Eq. (41), this procedure determines

$$\begin{aligned} C_A &= T_{\rm c,G}^{\alpha+1} - T_{{\rm c,fill},A}^{\alpha+1} = (\alpha+1)\left(\dot{Q}_0 + \dot{Q}_{{\rm NMR},A}\right)K \\ C_B &= T_{\rm c,G}^{\alpha+1} - T_{{\rm c,fill},B}^{\alpha+1} = (\alpha+1)\dot{Q}_0 K, \end{aligned} \tag{43}$$

where we use the fact that the heating due to pulse B is negligible. Then, the conversion of any

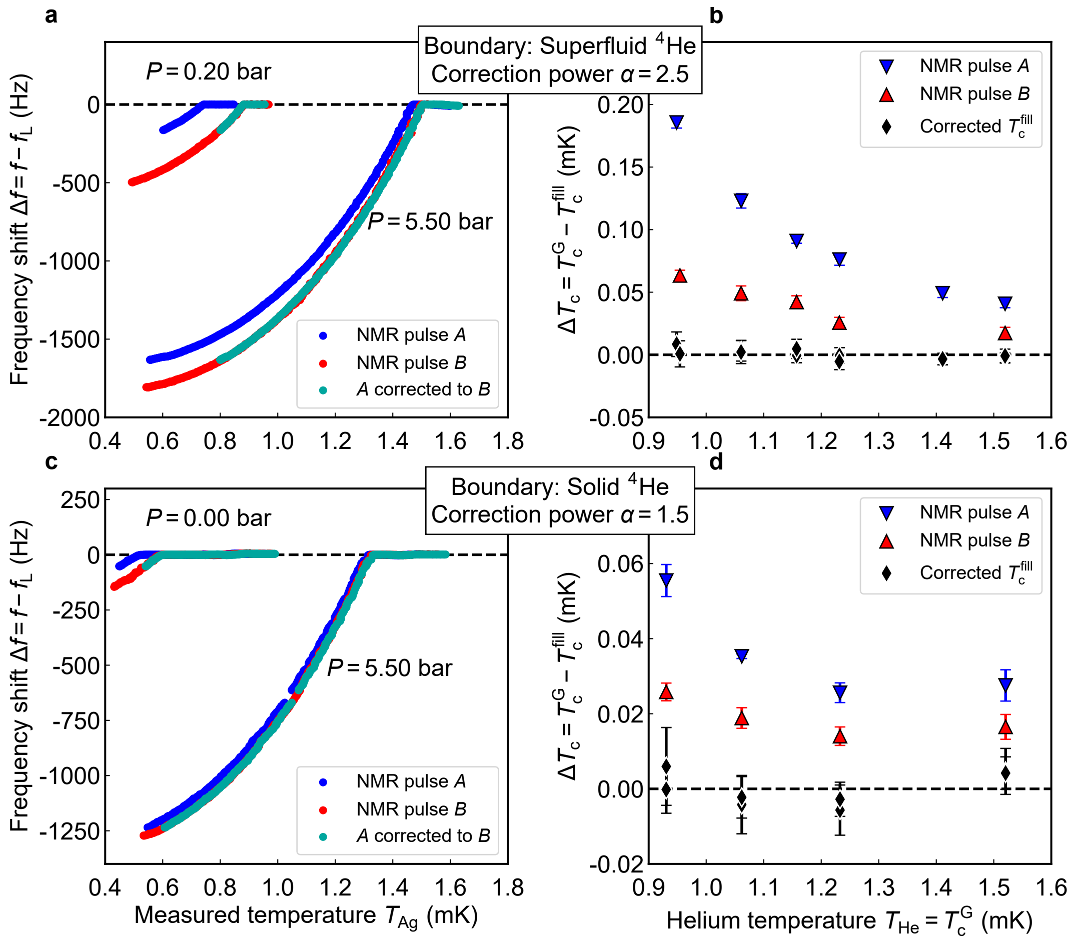

Supplementary Figure 13: Calibration of heating caused by NMR pulses. a, c Measured frequency shifts in the cavity at two pressures with "specular" (**a**) and "diffuse" (**c**) boundary conditions for NMR pulses A (blue circles) and B (red circles). Correction between pulses has been done using Supplementary Eq. (42) (cyan circles). **b, d** Difference between measured superfluid transition temperatures T_c^{fill} in the fill line bulk marker and the literature value T_c^{G} at different pressures corresponding to values of T_c^{G} between 0.9 and 1.6 mK. Each upwards and downwards pointing triangle represents the average of several measured bulk transitions with error bars indicating the experimental uncertainty. Corrected temperatures (diamonds) are reached using Supplementary Eq. (44), where increased error bars reflect the uncertainty of the correction.

measured temperature T_{Ag} to actual helium temperature T_{He} is

$$T_{\mathrm{He}} = \left(T_{\mathrm{Ag}}^{\alpha+1} + C_A\right)^{1/(\alpha+1)}, \tag{44}$$

with a similar expression for pulse B.

The results for C_A and C_B (Supplementary Fig. 14) show pressure independence. Therefore, the values are averaged over pressure and used to correct the bulk transition temperatures in

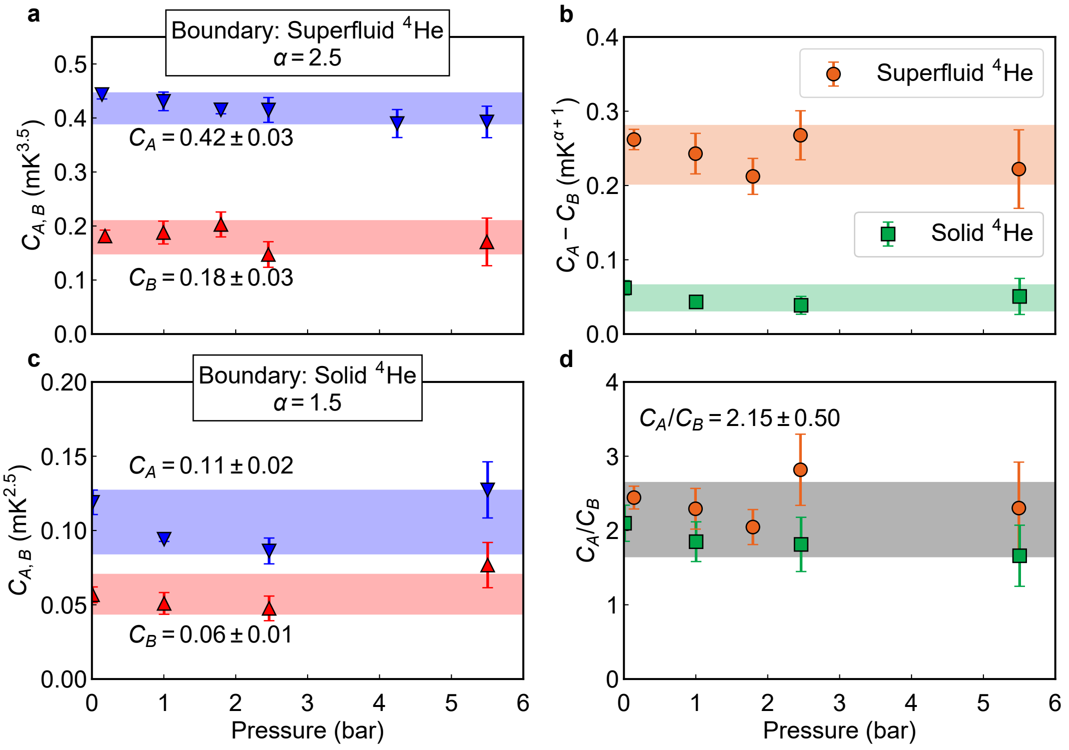

Supplementary Figure 14: Model parameters used in temperature correction. a, c Constants C_A (downwards pointing triangles) and C_B (upwards pointing triangles) (Supplementary Eq. (43)) with two boundary conditions as defined at bulk superfluid transitions in the fill line. Each symbol is an average based on several measured T_c^{fill}, same as in Supplementary Fig. 13b,d. The values of C_A and C_B are independent of pressure and significantly smaller with "diffuse" boundary condition due to smaller "poisoning" of heat exchanger by the thinner ^{4}He film. **b** Difference between constants C_A and C_B as a function of pressure with both boundary conditions, giving the correction between the pulses. **d** The ratio $C_A/C_B = 1 + \dot{Q}_{\mathrm{NMR},A}/\dot{Q}_0$ is defined from the same measurements of T_c^{fill} and does not depend on the boundary condition or the pressure, as expected. The coloured bands in each panel indicate the values averaged over pressure with the widths of the bands showing the uncertainties.

fill line as shown in Supplementary Fig. 13b,d. The usage of constant α and universal pressure-independent C_A or C_B satisfactorily corrects all the measurements within error limits from T_c^{G}. Similar correction has been applied to all reported temperatures in both the main text and the Supplementary Information. The uncertainties in C_A and C_B increase the error limits of the corrected temperatures as compared to the small errors in the original measured values. The lower the temperature is, the more dominant this is as a source of error. The systematic nature of this correction has been taken into account when calculating the overall error in relative temperatures T_c/T_{c0}.

We assume that the upper bound of the boundary resistance with a solid ^{3}He surface boundary layer is set by that of a solid ^{4}He layer in order to quantify uncertainties in temperature in this case.

SUPPLEMENTARY REFERENCES

[1] Zhelev, N. *et al.* Fabrication of microfluidic cavities using Si-to-glass anodic bonding. *Rev. Sci. Instrum.* **89**, 073902 (2018).

[2] Vollhardt, D. & Wölfle, P. *The superfluid phases of helium 3* (Dover Publications, Inc., Mineola, New York, 2013), Dover edn.

[3] Ahonen, A. I., Krusius, M. & Paalanen, M. A. NMR experiments on the superfluid phases of ^{3}He in restricted geometries. *J. Low Temp. Phys.* **25**, 421 (1976).

[4] Levitin, L. V. *et al.* Phase diagram of the topological superfluid ^{3}He confined in a nanoscale slab geometry. *Science* **340**, 841 (2013).

[5] Dmitriev, V. V. *et al.* Orbital glass and spin glass states of ^{3}He-A in aerogel. *JETP Lett.* **91**, 599 (2010).

[6] Rand, M. R. *Nonlinear spin dynamics and magnetic field distortion of the superfluid ^{3}He-B order parameter.* Ph.D. thesis, Northwestern University (1996).

[7] Rand, M. R. *et al.* New NMR evidence: Can an axi-planar superfluid ^{3}He-A order parameter be ruled out? *Physica B* **194-196**, 805 (1994).

[8] Schiffer, P., O'Keefe, M. T., Fukuyama, H. & Osheroff, D. D. Low-temperature studies of the NMR frequency shift in superfluid ^{3}He-A. *Phys. Rev. Lett.* **69**, 3096 (1992).

[9] Zimmerman, A. M., Nguyen, M. D. & Halperin, W. P. NMR frequency shifts and phase identification in superfluid ^{3}He. *J. Low Temp. Phys.* **195**, 358 (2019).

[10] Greywall, D. S. ^{3}He specific heat and thermometry at millikelvin temperatures. *Phys. Rev. B* **33**, 7520 (1986).

[11] Serene, J. W. & Rainer, D. The quasiclassical approach to superfluid ^{3}He. *Phys. Rep.* **101**, 221 (1983).

[12] Thuneberg, E. V. Hydrostatic theory of superfluid ^{3}He-B. *J. Low Temp. Phys.* **122**, 657 (2001).

[13] Zavjalov, V. V. ^{3}He library (2016). URL `http://github.com/slazav/he3lib`.

[14] Vorontsov, A. B. & Sauls, J. A. Thermodynamic properties of thin films of superfluid ^{3}He-A. *Phys. Rev. B* **68**, 064508 (2003).

[15] Vorontsov, A. B. Andreev bound states in superconducting films and confined superfluid ^{3}He. *Phil. Trans. R. Soc. A* **376**, 20150144 (2018).

[16] Sauls, J. A. Surface states, edge currents, and the angular momentum of chiral p-wave superfluids. *Phys. Rev. B* **84**, 214509 (2011).

[17] Nagato, Y., Higashitani, S., Yamada, K. & Nagai, K. Theory of rough surface effects on the anisotropic BCS states. *J. Low Temp. Phys.* **103**, 1 (1996).

[18] Nagato, Y., Yamamoto, M. & Nagai, K. Rough surface effects on the p-wave Fermi superfluids. *J. Low Temp. Phys.* **110**, 1135 (1998).

[19] Nagai, K., Nagato, Y., Yamamoto, M. & Higashitani, S. Surface bound states in superfluid ^{3}He. *J. Phys. Soc. Jpn.* **77**, 111003 (2008).

[20] Kjäldman, L. H., Kurkijärvi, J. & Rainer, D. Suppression of p-wave superfluidity in long, narrow pores. *J. Low Temp. Phys.* **33**, 577 (1978).

[21] Choi, H., Davis, J. P., Pollanen, J., Haard, T. M. & Halperin, W. P. Strong coupling corrections to the Ginzburg-Landau theory of superfluid ^{3}He. *Phys. Rev. B* **75**, 174503 (2007).

[22] Sauls, J. A. Private communication (2018).

[23] Ahonen, A. I. *et al.* Boundary magnetism in liquid ^{3}He at very low temperatures. *J. Phys. C* **9**, 1665 (1976).

[24] Godfrin, H. & Rapp, R. E. Two-dimensional nuclear magnets. *Adv. Phys.* **44**, 113 (1995).

[25] Freeman, M. R. & Richardson, R. C. Size effects in superfluid ^{3}He films. *Phys. Rev. B* **41**, 11011 (1990).

[26] Dyugaev, A. M. The theory of quantum nondegenerate liquids. *Sov. Sci. Rev. A Phys.* **14**, 1 (1990).

[27] Morhard, K.-D. *et al.* Two-dimensional Fermi liquid in the highly correlated regime: The second layer of ^{3}He adsorbed on graphite. *Phys. Rev. B* **53**, 2658 (1996).

[28] Lusher, C. P., Cowan, B. P. & Saunders, J. Quasiparticle interactions in two-dimensional fluid ^{3}He films adsorbed on graphite. *Phys. Rev. Lett.* **67**, 2497 (1991).

[29] Ramm, H., Pedroni, P., Thompson, J. R. & Meyer, H. Nuclear susceptibility of liquid He^3. *J. Low Temp. Phys.* **2**, 539 (1970).

[30] Bozler, H. M., Bates, D. M. & Thomson, A. L. Evidence for ferromagnetic order in the boundary

layer of ^{3}He on graphite. *Phys. Rev. B* **27**, 6992 (1983).

[31] Casey, A., Neumann, M., Cowan, B., Saunders, J. & Shannon, N. Two-dimensional ferromagnetism of a ^{3}He film: influence of weak frustration. *Phys. Rev. Lett.* **111**, 125302 (2013).

[32] Schuhl, A., Maegawa, S., Meisel, M. W. & Chapellier, M. Static and dynamic magnetic properties of ^{3}He confined by fluorocarbon microspheres. *Phys. Rev. B* **36**, 6811 (1987).

[33] Baker Jr., G. A., Gilbert, H. E., Eve, J. & Rushbrooke, G. S. On the two-dimensional spin-$\frac{1}{2}$ Heisenberg ferromagnetic models. *Phys. Lett.* **25A**, 207 (1967).

[34] Roger, M. High-temperature series expansions with cyclic exchanges on a triangular lattice: Application to two-dimensional solid ^{3}He. *Phys. Rev. B* **56**, R2928 (1997).

[35] Godfrin, H., Ruel, R. R. & Osheroff, D. D. Experimental observation of a two-dimensional Heisenberg nuclear ferromagnet. *Phys. Rev. Lett.* **60**, 305 (1988).

[36] Roger, M., Bäuerle, C., Bunkov, Yu. M., Chen, A.-S. & Godfrin, H. Multiple-spin exchange on a triangular lattice: A quantitative interpretation of thermodynamic properties of two-dimensional solid ^{3}He. *Phys. Rev. Lett.* **80**, 1308 (1998).

[37] Sauls, J. A., Bunkov, Yu. M., Collin, E., Godfrin, H. & Sharma, P. Magnetization and spin diffusion of liquid ^{3}He in aerogel. *Phys. Rev. B* **72**, 024507 (2005).

[38] Freeman, M. R., Germain, R. S., Thuneberg, E. V. & Richardson, R. C. Size effects in thin films of superfluid ^{3}He. *Phys. Rev. Lett.* **60**, 596 (1988).

[39] Eschrig, M. Scattering problem in nonequilibrium quasiclassical theory of metals and superconductors: General boundary conditions and applications. *Phys. Rev. B* **80**, 134511 (2009).

[40] Millis, A., Rainer, D. & Sauls, J. A. Quasiclassical theory of superconductivity near magnetically active interfaces. *Phys. Rev. B* **38**, 4504 (1988).

[41] Tokuyasu, T., Sauls, J. A. & Rainer, D. Proximity effect of a ferromagnetic insulator in contact with a superconductor. *Phys. Rev. B* **38**, 8823 (1988).

[42] Balatsky, A. V., Vekhter, I. & Zhu, J.-X. Impurity-induced states in conventional and unconventional superconductors. *Rev. Mod. Phys.* **78**, 373 (2006).

[43] Ovchinnikov, Yu. N. Critical current of thin films for diffuse reflection from the walls. *Sov. Phys. JETP* **29**, 853 (1969).

[44] Kopnin, N. B. Dynamics of superfluid helium-3 in flow channels with restricted geometries. *J. Low Temp. Phys.* **65**, 433 (1986).

[45] Sauls, J. A. & Sharma, P. Impurity effects on the $A_1 - A_2$ splitting of superfluid ^{3}He in aerogel. *Phys. Rev. B* **68**, 224502 (2003).

[46] Pollack, G. L. Kapitza resistance. *Rev. Mod. Phys.* **41**, 48 (1969).

[47] Pobell, F. *Matter and methods at low temperatures* (Springer-Verlag, Berlin, 1996), 2nd ed. edn.

[48] Rutherford, A. R., Harrison, J. P. & Stott, M. J. Heat transfer between liquid ^{3}He and sintered metal heat exchangers. *J. Low Temp. Phys.* **55**, 157 (1984).

[49] Autti, S. *et al.* Effect of the boundary condition on the Kapitza resistance between superfluid ^{3}He-B and sintered metal. *Phys. Rev. B* **102**, 064508 (2020).
